# Online Well-Being Group Coaching Program for Women Physician Trainees: A Randomized Clinical Trial

**DOI:** 10.1001/jamanetworkopen.2023.35541

**Published:** 2023-10-04

**Authors:** Adrienne Mann, Ami N. Shah, Pari Shah Thibodeau, Liselotte Dyrbye, Adnan Syed, Maria A. Woodward, Kerri Thurmon, Christine D. Jones, Kimiko S. Dunbar, Tyra Fainstad

**Affiliations:** 1 Division of Hospital Medicine, Department of Medicine, University of Colorado Anschutz Medical Campus, Aurora; 2 Veterans’ Health Administration, Eastern Colorado Health Care System, Aurora; 3 Division of Pediatric Surgery, Department of Surgery, Rush University Medical Center, Chicago, Illinois; 4 Department of Psychiatry, School of Medicine, University of Colorado Anschutz Medical Campus, Aurora; 5 Division of General Internal Medicine, Department of Medicine, University of Colorado Anschutz Medical Campus, Aurora; 6 University of Colorado School of Medicine, Aurora; 7 Veterans’ Health Administration, Eastern CO Health Care System, Denver-Seattle Center of Innovation for Veteran-Centered and Value Driven Care, Aurora, Colorado; 8 Perspectives Coaching Analytics LLC, Birmingham, Michigan; 9 Division of Urology, Department of Surgery, University of Colorado Anschutz Medical Campus, Aurora; 10 Division of Geriatrics, Department of Medicine, University of Colorado Anschutz Medical Campus, Aurora; 11 Children’s Hospital of Colorado, Section of Hospital Medicine, University of Colorado, School of Medicine, Aurora

## Abstract

**Importance:**

Physician burnout disproportionately affects women physicians and begins in training. Professional coaching may improve well-being, but generalizable evidence is lacking.

**Objective:**

To assess the generalizability of a coaching program (Better Together Physician Coaching) in a national sample of women physician trainees.

**Design, Setting, and Participants:**

A randomized clinical trial involving trainees in 26 graduate medical education institutions in 19 states was conducted between September 1, 2022, and December 31, 2022. Eligible participants included physician trainees at included sites who self-identified as a woman (ie, self-reported their gender identity as woman, including those who reported woman if multiple genders were reported).

**Intervention:**

A 4-month, web-based, group coaching program.

**Main Outcomes and Measures:**

The primary outcomes were change in burnout (measured using subscales for emotional exhaustion, depersonalization, and personal achievement from the Maslach Burnout Inventory). Secondary outcomes included changes in impostor syndrome, moral injury, self-compassion, and flourishing, which were assessed using standardized measures. A linear mixed model analysis was performed on an intent-to-treat basis. A sensitivity analysis was performed to account for the missing outcomes.

**Results:**

Among the 1017 women trainees in the study (mean [SD] age, 30.8 [4.0] years; 540 White participants [53.1%]; 186 surgical trainees [18.6%]), 502 were randomized to the intervention group and 515 were randomized to the control group. Emotional exhaustion decreased by an estimated mean (SE) −3.81 (0.73) points in the intervention group compared with a mean (SE) increase of 0.32 (0.57) points in the control group (absolute difference [SE], −4.13 [0.92] points; 95% CI, −5.94 to −2.32 points; *P* < .001). Depersonalization decreased by a mean (SE) of −1.66 (0.42) points in the intervention group compared with a mean (SE) increase of 0.20 (0.32) points in the control group (absolute difference [SE], −1.87 [0.53] points; 95%CI, −2.91 to −0.82 points; *P* < .001). Impostor syndrome decreased by a mean (SE) of −1.43 (0.14) points in the intervention group compared with −0.15 (0.11) points in the control group (absolute difference [SE], −1.28 (0.18) points; 95% CI −1.63 to −0.93 points; *P* < .001). Moral injury decreased by a mean (SE) of −5.60 (0.92) points in the intervention group compared with −0.92 (0.71) points in the control group (absolute difference [SE], −4.68 [1.16] points; 95% CI, −6.95 to −2.41 points; *P* < .001). Self-compassion increased by a mean (SE) of 5.27 (0.47) points in the intervention group and by 1.36 (0.36) points in the control group (absolute difference [SE], 3.91 [0.60] points; 95% CI, 2.73 to 5.08 points; *P* < .001). Flourishing improved by a mean (SE) of 0.48 (0.09) points in the intervention group vs 0.09 (0.07) points in the control group (absolute difference [SE], 0.38 [0.11] points; 95% CI, 0.17 to 0.60 points; *P* < .001). The sensitivity analysis found similar findings.

**Conclusions and Relevance:**

The findings of this randomized clinical trial suggest that web-based professional group-coaching can improve outcomes of well-being and mitigate symptoms of burnout for women physician trainees.

**Trial Registration:**

ClinicalTrials.gov Identifier: NCT05222685

## Key Points

**Question:**

Can a 4-month, online, group coaching program reduce burnout, moral injury, and impostor syndrome and increase self-compassion and flourishing among a sample of women physician trainees across multiple sites?

**Findings:**

In this randomized clinical trial of 1017 women trainee physicians, participants randomly assigned to a 4-month group-coaching program had a statistically significant reduction in all scales of burnout, moral injury, and impostor syndrome, as well as improved self-compassion and flourishing, compared with the control group.

**Meaning:**

These findings suggest that an online, multimodal, group coaching program is an effective intervention to decrease distress and improve well-being for women physician trainees.

## Teaser

This randomized clinical trial assesses whether an online group coaching program reduces burnout and increases well-being for women physician trainees.

## Introduction

Physician burnout is highly prevalent in the US; is disproportionately experienced by physician trainees and women; and is associated with substance abuse, job turnover, higher rates of medical errors, and patient mortality.^1,2,3,4,5^ In 2022, the US Surgeon General declared physician burnout a crisis deserving of a multipronged approach to bring about “bold, fundamental change,”^6^ yet little is known about scalable, effective interventions to mitigate burnout risk.^1,7,8,9^

Professional coaching is a promising intervention to reduce burnout.^10,11,12,13^ The 2022 Surgeon General’s Advisory emphasized building a culture of well-being in training institutions and included coaching as a recommended tool.^6,14^ Coaching, unlike therapy, does not diagnose or treat, and instead uses inquiry and metacognition (ie, thinking about one’s thinking) to guide self-progress.^15,16^ Evidence supporting physician coaching is growing, but predominantly describes individual coaching led by nonphysician or noncertified faculty coaches in small studies.^11,12,17,18,19,20^ Literature on outcomes of coaching for physician trainees is sparse, limited to small samples and single specialties, and primarily includes programs of short duration.^10,11,17,21,22^

An online group coaching program, Better Together Physician Coaching (BT), was piloted in response to high physician trainee burnout.^10,13^ Because women trainees are disproportionately affected by burnout,^3,4,5^ BT was initially evaluated among women resident physicians at the University of Colorado in a pilot, single site, randomized clinical trial (RCT), which indicated that online group coaching improved burnout.^10^ Building on previous work, the objective of this multisite RCT was to evaluate the generalizability of the 4-month online group coaching program to reduce distress and improve well-being in a national sample of women physician trainees.

## Methods

### Trial Oversight

This RCT follows the Consolidated Standards of Reporting Trials (CONSORT) reporting guideline^23^ and was approved by the University of Colorado institutional review board (see the Trial Protocol in Supplement 1). The study was conducted from September 1, 2022, to December 31, 2022, at 26 graduate medical education (GME) institutions across 19 states (eTable 1 in Supplement 2). Sites of different geographic locations, sizes, and focuses (ie, community vs academic) were recruited and included academic, county, Veterans Health Administration, and community-based hospitals and clinics. Recruitment emails were initially sent to institutional leadership, and video conference calls were held to confirm partnership in this study. Participant enrollment was voluntary, and all participants provided written informed consent. Data were collected and managed with The University of Colorado Research Electronic Data Capture (REDCap). Participating sites were not involved in research and could not access identifiable data.

### Participants and Trial Groups

All GME physician trainees who self-identified as a woman (ie, self-reported their gender identity as a woman, including those who reported woman if multiple genders were reported) at participating sites were eligible. Trainees were recruited through a series of 3 emails to a GME electronic mailing list. After enrollment, participants were randomly assigned to the intervention (access to online group coaching) or control group (no access). Randomization was stratified on the basis of the site. Intervention participants were not given protected time for the intervention and carried the same clinical schedules as control participants. All participants were asked to complete baseline (prior to randomization) and 4-month (end of intervention) surveys containing self-reported demographic questions and validated instruments measuring dimensions of distress and well-being. Participants had the ability to select multiple options for race and ethnicity and gender identity questions. Response options for gender identity included man, woman, nonbinary, not sure, not listed, and prefer not to say. Race and ethnicity categories included Asian, Black, Latinx, multiracial, Native American, Native Hawaiian and Other Pacific Islander, White, and other (defined as unknown or any other race or ethnicity not otherwise specified); race and ethnicity were included to ensure generalizability of the intervention. The control group was offered the intervention after the study. To account for benefits accruing from expectations rather than the intervention, both groups were emailed alternative online wellness resources.^24,25,26,27,28^

### Intervention: Better Together

BT is a 4-month, online, group coaching program developed by 2 professional physician coaches (T.F. and A.M.) and was delivered by a cohort of physician coaches (including author A.S.) who were all certified by The Life Coach School. Coach selection is described in the eMethods in Supplement 2. The program incorporates facets of user engagement from Short et al^29^ (eg, self-monitoring, reminders, and aesthetics), and the Cole-Lewis framework^30^ for behavior change (ie, modular, course-like, weekly introduction of content). More information on the foundational framework, program modalities, and curriculum can be found in the eMethods in Supplement 2. Participants had access to the following services housed on a members-only, password-protected website: (1) 3 to 4 live group coaching calls per week via video teleconference (Zoom Video Communications), (2) unlimited anonymous written coaching, and (3) weekly self-study modules on pertinent topics. Video teleconference sessions were recorded for asynchronous listening via private podcast. Topics were selected on the basis of an informal trainee needs assessment done prior to the pilot and were iteratively refined on the basis of rapid content analyses of coaching requests. The program required approximately 5 hours per week of total coach time.

### Distress Outcomes

#### Burnout

The 22-item Maslach Burnout Inventory (MBI) is considered the benchmark standard to measure burnout and was used under license.^31^ The MBI contains 3 subscales: emotional exhaustion (EE; score range, 0-54), where a higher score indicates worse EE; depersonalization (DP; score range, 0-30), where a higher score indicates worse DP; and personal accomplishment (PA; score range, 0-48), where a higher score indicates better feelings of PA.^32^ We used established threshold definitions of high EE (subscale score ≥27), high DP (subscale score ≥10), and low PA (subscale score ≤33), and considered those with high EE or DP to have at least 1 manifestation of burnout and meet the definition for positive burnout.^19,20,33^

#### Impostor Syndrome and Moral Injury

The Young Impostor Syndrome scale^34^ is an 8-item instrument with yes-or-no scoring and a range of 0 to 8. A score of 5 or greater indicates the presence of impostor syndrome. The Moral Injury Symptom Scale–Healthcare Professionals^35^ is a 10-item instrument with scores ranging from 10 to 100. Higher scores indicate greater moral injury.

### Well-Being Outcomes

#### Self-Compassion and Flourishing

The Neff Self-Compassion Scale–Short Form^36^ is a 12-item instrument (total scale range, 12-60). Scores of 12 to 30 are considered low, 30 to 42 are considered moderate, and 42 to 60 are considered high. The Secure Flourish Index^37^ is a 12-item instrument assessing 5 domains of flourishing. Scores range from 0 to12 and higher scores indicate greater flourishing.

### Statistical Analysis

We assumed no change in preintervention scores and postintervention scores for the control group and used estimates for the SD of the change in MBI scores from our pilot data.^10^ The power calculation for a minimum power of 80% (2-sided α = .05) to detect a standardized effect size (SD = 1) of 0.2, corresponded to a difference in mean (SD) values between the groups of 1.7 (8.6) in EE, 1.7 (8.6) in DP, and 1.0 (4.9) in PA, resulting in an enrollment target of 1000.

Descriptive statistics were computed for characteristics of the whole cohort and by intervention, with comparisons made using Wilcoxon rank sum tests for continuous covariates and Fisher exact or χ^2^ tests for categorical covariates. Characteristics of final survey responders and nonresponders were compared. Descriptive statistics were adjusted for multiple comparisons using the Benjamini-Hochberg procedure.^38^ *P* values from regression models were reported unadjusted.

An intent-to-treat analysis was performed on all participants regardless of postsurvey completion using linear and logistic mixed-effects models including the main effects of the time period (baseline vs postintervention), treatment (intervention vs control), the interaction between time period and treatment, and a random intercept for participants estimated using restricted maximum likelihood. Mean change from baseline within each group and the difference in mean change between groups and their 95% CIs were reported. Mixed-effects logistic regression models were used for EE, PA, DP, Young Impostor Syndrome Scale, Moral Injury Symptom Scale-Healthcare Professionals, Self-Compassion Scale-Short Form, Secure Flourish Index and for the binary outcomes of burnout and impostor syndrome.

Linear and logistic regression models were used to estimate mean change from baseline and odds ratios (ORs) adjusted for baseline value and their 95% CIs. The binary outcomes of burnout and impostor syndrome at follow-up were separately modeled using logistic regressions with treatment group and baseline values as variables. Effect sizes (risk difference, number needed to treat [NNT], and relative risk) for burnout and impostor syndrome were estimated from models using the same counterfactual framework for marginal effects. The 95% CIs were estimated with simulation.

In sensitivity analyses, to assess the potential influence of missing follow-up survey data on study outcomes, multiple imputation was used for missing score values via iterative chained random forest^39^ with predictive mean matching. The imputation model included baseline characteristics, treatment assignment, and baseline scores.

All *P* values are from 2-sided hypothesis tests and statistical significance was assessed at the α = .05 level. All analyses were performed using R statistical software version 4.2.3 (R Project for Statistical Computing).

## Results

### Participants

**Figure 1.**
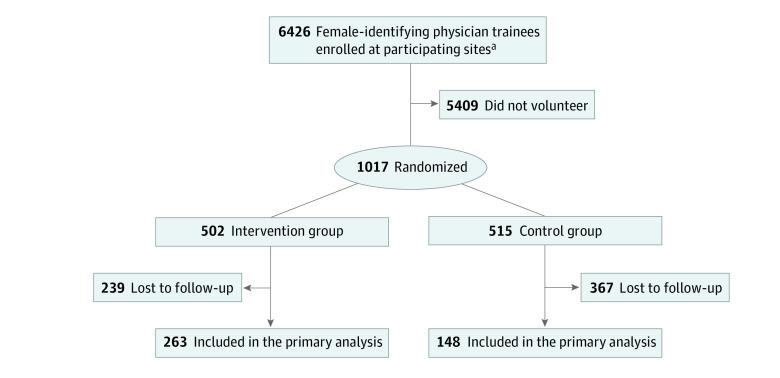
Participant Enrollment Flowchart

Of the 1017 participants (mean [SD] age, 30.8 [4.0] years; age range, 24.0-54.0 years; 540 White participants [53.1%]) enrolled, 502 were randomized to the intervention and 515 were randomized to the control group (Figure 1). All 26 sites had at least 1 participant in the intervention group (eTable 1 in Supplement 2). There were no significant baseline differences in demographics or outcome scores between groups (Table 1). Among all participants, 959 (94.3%) self-reported their gender identity as woman and 843 (88.0%) self-reported their sexual orientation as heterosexual. A total of 207 physician trainees (20.7%) were in postgraduate year (PGY) 1, 198 (19.8%) in PGY 2, and 595 (59.5%) in PGY 3 or beyond, with 186 of 999 physician trainees (18.6%) in a surgical subspecialty.

**Table 1.** Baseline Characteristics of All Enrolled Participants (Intention-to-Treat)

Variable or outcome	Participants, No. (%)		
	Overall (N = 1017)	Control (n = 515)	Intervention (n = 502)
Age, y			
Mean (SD)	30.9 (4.0)	31.0 (4.1)	30.8 (3.9)
Median (range)	30.0 (24.0-56.0)	30.0 (24.0-56.0)	30.0 (24.0-54.0)
Postgraduate year			
1	207(20.7)	98 (19.3)	109 (22.2)
2	198 (19.8)	97(19.1)	101 (20.5)
≥3	595 (59.5)	313 (61.6)	282 (57.3)
Training specialty			
Nonsurgical	813 (81.4)	417 (82.1)	396 (80.7)
Surgical, No./total No. (%)	186/999 (18.6)	91/508 (17.9)	95/491 (19.3)
Gender identity			
Woman	959 (94.3)	485 (94.2)	474 (94.4)
Man, nonbinary, other, or prefer not to say	58 (0.2)	29 (0.1)	28 (0.1)
Transgender			
No	938 (97.4)	476 (97.5)	462 (97.3)
Yes	24 (2.5)	11 (2.3)	13 (2.7)
Prefer not to say	1 (0.1)	1 (0.2)	0
Racial and ethnic identity			
Asian	229 (22.5)	110 (21.4)	119 (23.7)
Black	52 (5.1)	22 (4.3)	30 (6.0)
Latinx	83 (8.2)	41 (8.0)	42 (8.4)
Multiracial	35 (3.4)	20 (3.9)	15 (3.0)
Native American	2 (0.2)	0	2 (0.4)
Native Hawaiian and Other Pacific Islander	2 (0.2)	0	2 (0.4)
White	540 (53.1)	285 (55.3)	255 (50.8)
Other^a^	157 (15.4)	78 (15.1)	79 (15.7)
Sexual orientation, No./total No. (%)			
Heterosexual	843/958 (88.0)	433/487 (88.9)	410/471 (87.0)
Homosexual	21/958 (2.2)	11/487 (2.)	10/471 (2.1)
Bisexual	68/958 (7.1)	33/487 (6.8)	35/471 (7.4)
Other	6/958 (0.6)	4/487 (0.8)	2/471 (0.4)
Prefer not to say	20/958 (2.1)	6/487 (1.2)	14/471 (3.0)
Distress outcome			
Burnout			
MBI personal achievement subscale			
Completed subscale, No. overall/No. observed (% not missing)	812 (79.8)	405(78.6)	407 (81.1)
Score, mean (SD)	33.3 (7.3)	33.5 (7.2)	33.1 (7.3)
Score, Median (IQR)	34.0 (29.0-38.0)	34.0 (29.0-38.0)	34.0 (28.0-38.0)
Score, Range	8.0-48.0	11.0-48.0	8.0-47.0
MBI emotional exhaustion subscale			
Completed subscale, No. overall/No. observed (% not missing)	788 (77.5)	394 (76.5)	394 (78.5)
Score, mean (SD)	30.6 (10.8)	30.5 (11.0)	30.6 (10.5)
Score, median (IQR)	31.0 (23.0-38.0)	32.0 (23.0-39.0)	31.0 (23.0-38.0)
Score, range	8.0-48.0	11.0-48.0	8.0-47.0
MBI depersonalization subscale			
Completed subscale, No. overall/No. observed (% not missing)	810 (79.6)	405 (78.6)	405 (80.7)
Score, mean (SD)	11.8 (6.4)	11.8 (6.6)	11.9 (6.3)
Score, median (IQR)	12.0 (7.0-17.0)	12.0 (6.0-17.0)	11.0 (7.0-16.0)
Score, range	0.0-29.0	0.0-29.0	0.0-28.0
Met definition for positive burnout, No. overall/No. observed (% not missing)			
No	211/781 (27.0)	108/391 (27.6)	103/390 (26.4)
Yes	570/781 (73.0)	283/391 (72.4)	287/390 (73.6)
Impostor syndrome			
Completed YIS, No. overall/No. observed (% not missing)	784/1017 (77.1)	388/515 (75.3)	396/502 (78.9)
YIS score, mean (SD)	5.8 (2.0)	5.8 (1.9)	5.9 (2.0)
YIS score, median (IQR)	6.0 (5.0-7.2)	6.0 (5.0-7.0)	6.0 (5.0-8.0)
YIS score, range	0.0-8.0	0.0-8.0	0.0-8.0
YIS ≥5			
No	186/784 (23.7)	91/388 (23.5)	95/396 (24.0)
Yes	598/784 (76.3)	297/388 (76.5)	301/396 (76.0)
Moral injury			
Completed MISS-HP	787(77.4)	393 (76.3)	394 (78.5)
MISS-HP score, mean (SD)	45.9 (13.7)	46.6 (13.8)	45.2 (13.6)
MISS-HP score, median (IQR)	47.0 (36.0-55.0)	47.0 (37.0-56.0)	45.5 (35.0-54.0)
MISS-HP score, range	12.0-95.0	12.0-95.0	15.0-89.0
Well-being outcome			
Self-compassion			
Completed SCS-SF, No. overall/No. observed (% not missing)	788 (77.5)	391 (75.9)	397 (79.1)
SCS-SF score, mean (SD)	31.6 (7.3)	31.7 (7.4)	31.4 (7.2)
SCS-SF score, median (IQR)	31.0 (27.0-36.0)	31.0 (27.0-36.0)	31.0 (26.0-37.0)
SCS-SF score, range	12.0-60.0	12.0-60.0	14.0-55.0
Flourishing			
Completed SFI, No. overall/No. observed (% not missing)	737 (72.5)	366(71.1)	371 (73.9)
SFI score, mean (SD)	6.2 (1.3)	6.2 (1.3)	6.2 (1.3)
SFI score, median (IQR)	6.2 (5.2-7.2)	6.2 (5.2-7.1)	6.2 (5.2-7.2)
SFI score, range	2.6-9.5	3.0-9.5	2.6-9.5

Abbreviations: MBI, Maslach Burnout Inventory; MISS-HP, Moral Injury Symptom Scale-Healthcare Professionals; SCS-SF, Self-Compassion Scale-Short Form; SFI, Secure Flourish Index; YIS, Young Imposter Syndrome scale.

^a^ Other race and ethnicity was defined as unknown or any other race or ethnicity not otherwise specified.

At baseline, the mean (SD) score was 30.6 (10.8) for EE, 11.8 (6.4) for DP, and 33.3 (7.3) for PA. A total of 570 participants scored positively for burnout, which represents 73.0% of the 781 participants who completed the full MBI. Of 784 participants who completed the YIS, 598 (76.3%) scored positively for impostor syndrome. The mean (SD) score was 45.9 (13.7) for moral injury, 31.6 (7.3) for self-compassion, and 6.2 (1.3) for flourishing. There were 411 participants (40.4%) who responded to the follow-up survey, with more participants in the control group (263 participants [51.0%; 95% CI, 47%-55%]) than the intervention group (148 participants [30.0%; 95% CI, 26%-34%]) (P < .05). Postsurvey respondents and nonrespondents differed by race, gender identity, and PGY (eTable 2 in Supplement 2).

### Distress Outcomes

#### Burnout

**Figure 2.**
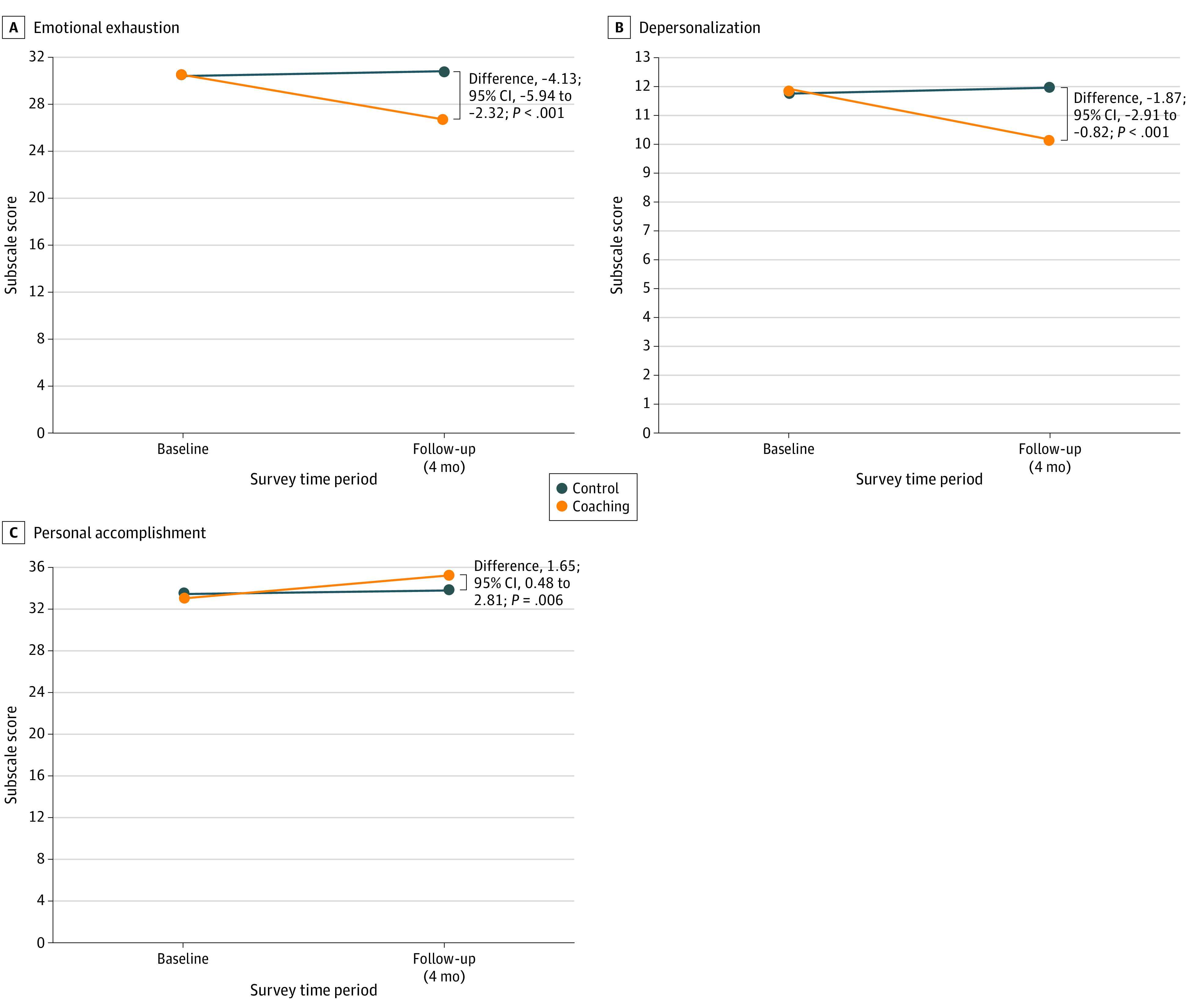
Mean Change in Burnout Response From Baseline Visit, Estimated From Linear Mixed-Effects Models The figure shows the change in score for both the control group and intervention group from baseline to follow-up on the 3 subscales of the Maslach Burnout Inventory: emotional exhaustion (A), depersonalization (B), and personal accomplishment (C).

Intervention participants experienced significant improvement compared with the control group on all MBI subscales. For EE, the estimated change in score for the intervention group (mean [SE] −3.81 [0.73] points; 95% CI, −5.24 to −2.38 points) vs the control group (mean [SE] 0.32 [0.57] points; 95% CI, −0.79 to 1.43 points) resulted in an absolute difference (SE) in EE of −4.13 (0.92) points (95% CI, −5.94 to −2.32 points; *P* < .001).For DP, the estimated change in score for the intervention group (mean [SE] −1.66 [0.42] points; 95% CI, −2.49 to 0.83 points) vs the control group (mean [SE] 0.20 [0.32] points; 95% CI, −0.43 to 0.84 points) resulted in an absolute difference (SE) of −1.87 (0.53) points (95% CI, −2.91 to −0.82 points; *P* < .001). For PA, the estimated change in score for the intervention group (mean [SE] 2.08 [0.47] points; 95% CI, 1.15-3.00 points) vs control group (mean [SE] 0.43 [0.36] points; 95% CI, −0.28 to 1.13 points) resulted in an absolute difference (SE) of 1.65 (0.59) points (95% CI, 0.48 to 2.81 points; *P* = .006) (Table 2 and Figure 2).

**Table 2.** Mean Change in Response From Baseline Visit, Established From Linear Mixed-Effects Models

Outcome	Intervention group		Control group		Absolute difference (SE) in score change for intervention vs control, points [95% CI]	*P* value
**Participants, No.**	**Estimated score change, points (SE) [95% CI]**	**Participants, No.**	**Estimated change, points (SE) [95% CI]**
Distress						
Burnout						
MBI emotional exhaustion subscale score						
Baseline	394	−3.81 (0.73) [95% CI, −5.24 to −2.38]	394	0.32 (0.57) [−0.79 to 1.43]	−4.13 (0.92) [−5.94 to −2.32]	<.001
After intervention	142		256			
MBI depersonalization subscale score						
Baseline	407	−1.66 (0.42) [−2.49 to 0.83 points]	405	0.20 (0.32) [−0.43 to 0.84]	−1.87 (0.53) [−2.91 to −0.82]	<.001
After intervention	144		260			
MBI personal achievement subscale score						
Baseline	405	2.08 (0.47) [1.15-3.00]	405	0.43 (0.36) [−0.28 to 1.13]	1.65 (0.59) [0.48 to 2.81]	.006
After intervention	143		260			
Young Impostor Syndrome scale score						
Baseline	396	−1.43 (0.14) [−1.70 to −1.15]	388	−0.15 (0.11) [−0.36 to 0.07]	−1.28 (0.18) [−1.63 to −0.93]	<.001
After intervention	140		252			
Moral Injury Symptom Scale score						
Baseline	394	−5.60 (0.92) [−7.40 to −3.81]	393	−0.92 (0.71) [−2.31 to 0.46]	−4.68 (−2.41) [−6.95 to 2.41]	<.001
After intervention	142		254			
Well-being						
Self-compassion Scale-Short Form score						
Baseline	397	5.27 (0.47) [4.34 to 6.20]	391	1.36 (0.36) [0.65 to 20.80]	3.91 (0.60) [2.73 to 5.08]	<.001
After intervention	142		255			
Secure Flourish Index score						
Baseline	371	0.48 (0.09) [0.31 to 0.65]	366	0.09 (0.07) [−0.04 to 0.23]	0.38 (0.11) [0.17 to 0.60]	<.001
After intervention	131		240			

Abbreviation: MBI, Maslach Burnout Inventory.

Intervention participants experienced an 18% (95% CI, 5%-31%) reduction in probability of burnout, with an OR of 0.47 (95% CI, 0.28-0.78; *P* = .008) compared with the control (ie, intervention participants had 53% lower odds of experiencing burnout at follow-up), with an NNT of 11 (95% CI, 7.1-22.4) to go from positive to negative burnout (eTable 3 in Supplement 2).

#### Impostor Syndrome

**Figure 3.**
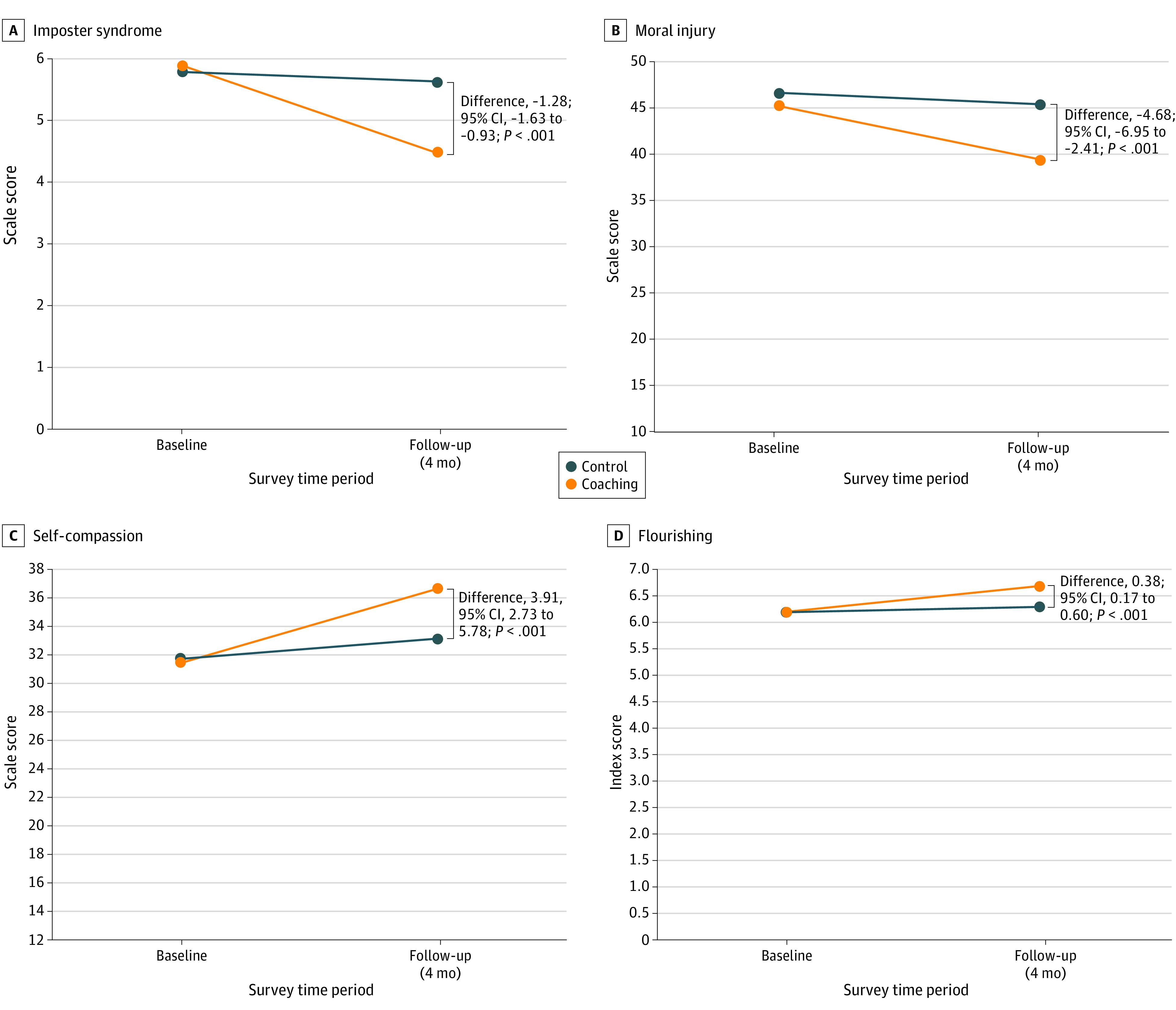
Mean Change in Secondary Outcome Response From Baseline Visit, Estimated From Linear Mixed Effects Models The figure shows the change in score for both the control group and intervention group from baseline to follow-up for Impostor syndrome (A), moral injury (B), self-compassion (C), and flourishing (D).

Intervention participants experienced a significant decrease in impostor syndrome with an absolute difference (SE) of −1.28 (0.18) points (95% CI, −1.63 to −0.93 points; *P* < .001); the mean (SE) change in score for the intervention group was −1.43 (0.14) points (95% CI, −1.70 to −1.15 points) and the mean (SE) change in score for the control group was −0.15 (0.11) points (95% CI, −0.36 to 0.07 points) (Figure 3). Intervention participants had a 34% (95% CI, 16% to 52%) reduced probability of impostor syndrome (OR, 0.36; 95% CI, 0.21 to 0.62; *P* < .001) with an NNT of 9 (95% CI, 6.8 to 17.8 to go from positive to negative impostor syndrome (eTable 3 in Supplement 2).

#### Moral Injury

Intervention participants experienced a significant decrease in moral injury with an absolute difference (SE) of −4.68 (1.16) points (95% CI, –6.95 to −2.41 points; *P* < .001). The mean (SE) change on the Moral Injury Symptom Scale for the intervention group was −5.60 (0.92) points (95% CI, −7.40 to −3.81 points) vs −0.92 (0.71) points (95% CI, −2.31 to 0.46 points) for the control group (Figure 3).

### Well-Being Outcomes

#### Self-Compassion

There was a significant improvement in self-compassion with an absolute difference (SE) of 3.91 (0.60) points (95% CI, 2.73 to 5.08 points; *P* < .001). The mean (SE) change on the Self-Compassion Scale–Short Form was 5.27 (0.47) points (95% CI, 4.34 to 6.20 points) for the intervention group vs 1.36 (0.36) points (95% CI, 0.65 to 20.80 points) for the control group (Figure 3).

#### Flourishing

There was a significant improvement in flourishing with an absolute difference (SE) of 0.38 (0.11) points (95% CI, 0.17 to 0.60 points; *P* < .001). The mean (SE) change on the Secure Flourish Index was 0.48 (0.09) points (95% CI, 0.31 to 0.65 points) for the intervention group vs 0.09 (0.07) points (95% CI, −0.04 to 0.23 points) for the control group (Figure 3).

### Sensitivity Analysis

Similar outcome results were obtained using multiple imputation and when baseline scores were carried forward for missing follow-up scores. See eTable 4 in Supplement 2 for more information on the sensitivity analysis.

## Discussion

In this large, national RCT, women physician trainees who received online group coaching over 4 months had substantial reductions in multiple dimensions of professional distress (burnout, moral injury, and impostor syndrome) and improvements in well-being (self-compassion and flourishing). The improvement in burnout was significant and likely meaningful, with past studies^40,41^ showing that even a 1-point increase in EE has been associated with a 7% increase in suicidal ideation and a 5% increase in self-reported major medical errors. Additionally, intervention trainees reported less moral injury, lower odds of impostor syndrome, and increased flourishing and self-compassion.

We have previously demonstrated online group coaching reduced burnout among women trainees across specialties at a single institution.^10^ The digital platform and group nature of this study allowed a 10-fold expansion of participants and an addition of 25 sites with only 30 additional hours of direct coaching (1-2 more calls per week compared with the pilot study).^10^ The group and online delivery of the intervention supported greater scalability, accessibility, and lower cost compared with individual coaching. Furthermore, the magnitudes of improvement in scores described here were generally higher than other coaching and wellness interventions^17,19,20,42,43,44^ and higher than that of our single-site pilot RCT.^10^ Our findings show effectiveness in a broader population and match national data for trainees that self-report as underrepresented in medicine.^45^ We hypothesize that the greater impact may be due to iterative refinements, the maturity of the program, and the addition of nationwide participants who created community and normalized otherwise isolating challenges in medicine.^13^

In a recent advisory,^46^ the US Surgeon General strongly recommends integrating social connection into wellness programs focused on burnout. A major theme that arose from a qualitative analysis^13^ of participants’ experience of online group coaching was an increased sense of connection, even during a time of profound social isolation during the COVID-19 pandemic. Given the digital platform, online group coaching is easily accessed by rural and less resourced programs. Online group coaching is an example of an institutionally provided, individually harnessed tool to build a culture of connection necessary to heal physician burnout.

### Limitations

This study has limitations. Voluntary participation may have created selection bias. This study enrolled only participants who identify as women, so outcomes among individuals who identify as men is unknown. There could have been selection bias of sites, a process that was dependent on leadership buy-in, which is vulnerable to bias. We did not use cluster randomization given our interest in individual rather than group-level changes in outcomes, and we also wanted to avoid loss of statistical power. This study is unique in that the intervention was administered digitally by a centralized entity and not separately at each site as in more traditional clinical trials. However, we may consider cluster randomization in future studies. Contamination was possible because each site had participants in control and intervention groups, although mitigation efforts were implemented (eg, password access to intervention materials).

We had substantial loss to follow-up. Control participants were significantly more likely to respond to the survey than intervention participants, perhaps due to email fatigue (the intervention group received 2emails weekly), or control participants may have been more motivated in anticipation of receiving the intervention. There were racial, gender identity, and PGY differences between follow-up survey responders and nonresponders. This finding may be related to previously established observations that those with higher time constraints (ie, participants in PGY 1) may feel marginalized, and those who are underrepresented in medicine due to their race and/or gender identity may feel less socially connected and less inclined to complete surveys.^47,48^ We attempted to assess the effect of missing follow-up survey data on study outcomes with the sensitivity analysis, a statistical technique to evaluate the effect of the missing data (eTable 4 in Supplement 2), which did not find significant outcome differences. Because participants did not use the same login for each coaching call, downloaded podcast, or curriculum module, we were unable to measure engagement or correlate it with outcomes. Additionally, the study team and participants could not be masked, and outcomes could have accrued in part from participant expectations despite providing both groups access to well-being resources as a plausible alternative. Furthermore, we did not evaluate the postintervention effect, which warrants future study.

## Conclusions

Compared with GME training as usual, an online 4-month group-coaching program for women physician trainees delivered by certified physician coaches resulted in significant improvement in professional distress and well-being, with an NNT of 11 to mitigate burnout. Group coaching interventions may have a role in mitigating physician trainee burnout and improving well-being along with system-level interventions to improve the work and learning environment.

## Notes

Supplement 1.
Trial Protocol

Supplement 2. **eTable 1.** Site Participation **eMethods**. BT Coach Onboarding and Facets of BT Program **eTable 2.** Participant Characteristics at Baseline by Survey Completion **eTable 3.** Effect Sizes for Burnout and Impostor Syndrome **eTable 4.** Intention to Treat Sensitivity Analysis: Mean Change in Response From Baseline Visit, Estimated From Linear Regression Adjusted for Baseline Value

Supplement 3.
Data Sharing Statement
