# Supplementary material for: Online Well-Being Group Coaching Program for Women Physician Trainees: A Randomized Clinical Trial
Source: JAMA Netw Open. 2023 Oct 4;6(10):e2335541. doi: 10.1001/jamanetworkopen.2023.35541 (PMC10551770; doi:10.1001/jamanetworkopen.2023.35541)
Supplement: Supplement 1. — Trial Protocol [file jamanetwopen-e2335541-s001.pdf]

Online Well-Being Group Coaching Program for Women Physician Trainees: A Randomized Clinical Trial

This Supplement contains:

1. Original protocol, final protocol, and summary of changes to the protocol
2. Statistical analysis plan (SAP)

COMIRB Protocol

COLORADO MULTIPLE INSTITUTIONAL REVIEW BOARD

CAMPUS BOX F-490 TELEPHONE: 303-724-1055 Fax: 303-724-0990

ORIGINAL STUDY PROTOCOL

Protocol #: 22-0028

Project Title: Better Together Physician Coaching: An Innovative Solution to Medical Trainee Burnout

Principal Investigator: Dr. Tyra Fainstad
tyra.fainstad@cuanschutz.edu, 720-480-4970

Version Date: January 6th 2022

I. Hypotheses and Specific Aims: Our HYPOTHESIS is that Better Together, a 6-month, web-based positive psychology coaching program intervention for female residents when expanded to GME programs nationally, will result in decreased female trainee burnout.

· Aim 1: Prepare to expand the Better Together program by training and hiring a cohort of diverse physician coaches

· Aim 2: Implement Better Together in 10+ GME programs to serve 1000+ trainee participants with deliberate inclusion of institutions with diverse GME trainee populations serving geographically rural and/or medically underserved areas.

· Aim 3: Assess our outcomes: primary: reduce burnout as measured by the Maslach Burnout Index (goal: 10% relative improvement), and secondary: self-compassion, imposter syndrome and moral injury. We will also analyze outcome generalizability and program feasibility at a national level and look qualitatively at participant experience to gain a richer understanding of how Better Together may help trainees, in particular underrepresented minorities.

· Aim 4: Advance the field of coaching in GME through innovation and dissemination of evidence-based approaches to GME trainee wellbeing.

II. Background and Significance:

Burnout refers to feelings of exhaustion, negativism, and reduced personal efficacy resulting from chronic workplace stress. In healthcare, burnout leads to increased medical errors, poorer patient care and negatively affects professional development and retention. Burnout is a growing problem that begins early in medical training. Women and those underrepresented in medicine (URM) experience a disproportionate amount of burnout likely due to the cognitive load required to manage microaggressions, stereotypes, and harmful socially adopted narratives around efficacy. Professional coaching is a metacognition tool with a sustainable positive effect on physician well-being but typically relies on expensive consultants or time-

consuming faculty development, often making it infeasible for medical training programs to offer. To overcome this barrier, we created Better Together Physician Coaching (BT) a 6-month coaching program for women residents at the University of Colorado (CU). BT includes regular online group-coaching, written coaching, and weekly self-study modules delivered by physician life coaches (Co-PIs). A pilot randomized controlled trial (RCT) of 101 BT participants demonstrated a statistically significant improvement in burnout, self-compassion, and imposter syndrome in the intervention group. This project will scale Better Together up to a national level and evaluated with an RCT mirroring our pilot in 10+ graduate medical education (GME) programs for 1000+ participants coordinated and evaluated by our CU team.

III. Preliminary Studies/Progress Report:

Within the past year, Co-PIs Drs. Adrienne Mann and Tyra Fainstad (AM, TF) created and implemented Better Together Physician Coaching (Better Together, or BT) at the University of Colorado School of Medicine. BT is a coaching program for female identifying GME trainees at CU and its affiliate hospitals (Denver Health, Rocky Mountain Regional VA Medical Center, and The Children's Hospital). Using coaching techniques, BT challenges long-held paradigms fostered by medical training. BT aims primarily to reduce burnout as measured by the MBI with secondary aims to increase self-compassion, reduce imposter syndrome and decrease moral injury among residents who identify as women.

The Co-PIs (AM, TF) are both certified coaches through The Life Coach School™, a training institution for thought-based coaching. This type of coaching focuses on thoughts and beliefs. It combines a cognitive behavioral therapy (CBT) model with mindfulness-based awareness and integrates theories of acceptance and commitment therapy (ACT), nonattachment, and radical questioning from Socratic and Greek philosophies. BT delivers a robust coaching experience via a 6-month web-based, group-coaching model. This novel program allows residents to participate as actively as they are inclined and able, offering flexibility via multiple modalities of coaching: twice weekly group coaching calls, unlimited anonymous written coaching, and weekly self-study modules that are housed on a secure members-only website.

To study the BT program, the Co-PIs received institutional support from the CU Department of Medicine to conduct a pilot randomized controlled trial (RCT), which included support for professional research assistants to both implement and evaluate the program. A convenience sample of 101 female-identifying CU GME trainees from 12 specialties (IM, Family Medicine, Otolaryngology, Pediatrics, OBGYN, General Surgery, Emergency Medicine, Dermatology, Psychiatry, Medicine-Pediatrics, Pathology, and Neurology) was recruited and randomized to receive the 6-month Better Together Program or no-intervention from January-June 2021. The median participant age was 29 years, and all were female-identifying. Of the 101 participants, 33 (32.7%) were PGY-1, 43 (42.6%) were PGY-2, 18 (17.8%) were PGY-3, and 7 (7%) PGY-4 or greater. Nineteen (19%) of participants were in a surgical residency specialty (general surgery, OBGYN, otolaryngology). There were no significant differences in these characteristics between the intervention and control groups at baseline.

All participants completed a pre-survey assessing burnout with the MBI which defines burnout by three subscales (Emotional Exhaustion (EE), Depersonalization (DP), and Personal Accomplishment (PA) as described above). Each item is a 7-point Likert-type question with a frequency response scale ranging from 0 = never to 6 = every day. Higher scores on the EE and DP, and lower scores on the PA subscales indicate greater burnout. Secondary outcomes included Self-compassion with Self-Compassion Scale Short-form (SCSS) where higher scores indicate greater self-compassion,30 the Young Imposter Syndrome Scale (YISS) where a score

of more than 5 out of 8 points is diagnostic for Imposter Syndrome31 and the Moral Injury Symptom Scale (MISS) (score 10-100 points) where higher scores equal more moral injury.32

Participants were randomized to the intervention group, (N=50) or control group (N= 51). The intervention group received the 6-month BT coaching program. The control group received the usual wellbeing curriculum provided by their training program, but no BT intervention. Within the BT coaching group, the most frequent topics of coaching included feedback reception, professional appearance, approval-addiction, deficit hiding, balancing motherhood with residency, and low self-confidence. Participants engaged in the live coaching sessions with curiosity, vulnerability, and authenticity, often bringing highly personal and emotional issues to the sessions and supporting each other through encouragement in the Zoom chat function.

From the pilot RCT, we found that at baseline over half of all participants were experiencing moderate or high burnout, consistent with national data. Participants were also experiencing low-moderate self-compassion (mean = 33.6 out of 60; SD=7.17); imposter syndrome (mean = 5.4 out of 8, where score of 5+ is diagnostic for imposter syndrome; SD=2.13); and moderate moral injury (mean =42.02 out of 100; SD=11.08). At the end of 6 months of coaching, a post-survey was offered to both the intervention and control groups. Of the 101 initial survey respondents, 79 responded to the post-survey (78%). A t-test was used to compare the change in subscale score means from baseline to 6 months in the BT coaching versus control groups for the primary and secondary outcomes.

The results on the MBI showed a statistically significant decrease in the emotional exhaustion (EE) dimension of burnout in the intervention group (p=0.03), and the DP and PA components of burnout both trended toward improvement. Self-compassion improved significantly in the intervention group compared to the control group, and imposter syndrome scores improved in the BT coaching group from 5.4 to 4.2 (p=0.01), effectively improving mean scores out of the range for imposter syndrome in this group. Moral injury also trended towards an improvement in the coaching vs control group from baseline to 6 months (40.7 to 35.6 versus 43.7 to 41.7 in intervention vs control, mean difference -3.84, p 0.10), but was not statistically significant.

Based on these promising findings in our pilot RCT, we propose scaling Better Together to a national level to address the unmet need in GME programs to reduce burnout. We have applied for HRSA grant funding (pending) which will allow us to plan, implement, and study a multi-institutional RCT to serve more female identifying trainees with a focus on those who are URM and practicing in medically underserved communities nationwide. In addition to adding an evidence-based innovative program to improve trainee burnout and wellness, this expanded trial will examine its generalizability and the feasibility of a model in which new coaches are trained to implement BT through a train-the-trainer model. We aim to enroll GME programs with geographic and ethnic diversity as well as representative URM trainees to understand the impact of coaching in this vulnerable population.

IV. Research Methods

A. Outcome Measure(s):

The primary outcome measure will include measures of burnout using the Maslach Burnout Inventory (MBI). Additional outcomes will include measures of self-compassion, imposter syndrome, and moral injury, as well as participation (administrative data), and participant experience and reflection (interview). All data collection will be done in an aggregated format and in a confidential manner.

B. Description of Population to be Enrolled:

We will use convenience sampling to recruit an average of 100 trainee participants per GME program site, for a total of up to 2000 trainee participants across up to 20 GME programs. Confirmed sites include the University of Washington, Johns Hopkins, St. Joseph's/Kaiser Colorado, Rush, Nuvance Residency Program, Cooper University, University of California San Diego, Brook Army Medical Center, University of Kentucky, Kansas University Medical Center, Providence Eastern Washington, Tufts, Virginia Mason, Providence Alaska, and University of Texas San Antonio. Inclusion criteria are: GME trainees (residents and fellows) who identify as women, trans-women, transfeminine, gender nonconforming, non-binary, and gender queer, who have more than 1 year left in their training program. The rationale for this last criterion is to ensure enrolled trainees will have time to complete the program prior to graduation. Enrollment in the program will be entirely voluntary and trainees can cease enrollment at any time.

C. Study Design and Research Methods

In this randomized controlled trial, we will use a Plan-Do-Study-Act (PDSA) approach. This will be done in 2 "rounds". For each round, the intervention group will receive BT during the first 6-months while the control group, or "waitlist group" will receive no intervention aside from the usual wellness activities of their institution.

Round 1:

Round 1 will begin in September 2022 with approximately 500-1000 trainees from 8-10 institutions selected at random from the 12 participating sites. Following the pre-survey, participants will be randomized to the intervention group or the control group. The intervention group will receive the 6-month BT program from 9/2022-2/2023. All participants will complete the post survey in 2/2023. Participants in the intervention group will be offered opportunities to complete qualitative interviews around the program experience after completion of the program. The control group will then be offered the Better Together program from 3/2023 - 9/2023. Following a 1-month interlude, round 2 will follow the same structure of Round 1, with recruitment beginning March 2023 through Sept 2023, and program implementation occurring from October 2023 through March of 2024.

The BT coaching program will be led by AM with support from KT as the lead-coach. KT will coordinate the weekly schedule of coaching activities among the group of BT coaches (includes AM, TF, KT who are already certified, and the CCs who will have completed certification). The coach consultants will each provide 1-2 coaching calls per week as well as focused workshops, seminars, and panels as needed.

Evaluation of Round 1 will be led by TF in partnership with CJ, TEC and the PRA. During this 1-month interlude (9/2023), the evaluation team will schedule and begin to conduct 1:1 qualitative interviews, compile the quantitative data, and begin to code the qualitative data. The pre-survey will be administered to Round 2 participants prior to randomization. Revisions to the BT program content and structure will be informed in PDSA style via inputs from quantitative and qualitative trainee data as well as feedback and impressions from the BT coaching team.

Round 2:

Round 2 will begin in October 2023 with ~500-1000 participants from the remaining 8-10 institutions. Implementation will mirror Round 1 and will incorporate changes guided by prior PDSA cycles. The Round 2 intervention group will receive BT from 10/2023-3/2024. All Round 2 participants will complete the post-survey in 3/2024. The control group will receive BT from 4/2024-9/2024. BT program content, delivery and evaluation plan will mirror that as outlined above for round 1.

Wrap-up:

The final 3 months will focus on preparing our final results for dissemination and finalizing our sustainability plan as we prepare for our future planned funding model.

D. Description, Risks and Justification of Procedures and Data Collection Tools:

This is a low-risk intervention. Positive psychology coaching is not meant to replace or function as evaluation or medical care. We are using an internally developed survey in addition to multiple validated surveys that reflect the literature in similar programs. Any participant who demonstrates medically concerning issues will be immediately referred to appropriate evaluation. The content of the program is not intended for evaluation, so will not be shared with training programs. The participants will be instructed to maintain confidentiality of their peers' information, although given the group nature of this intervention, confidentiality cannot be assured. The faculty interests in their roles as clinical supervisors and coach are aligned in the promotion of trainee success. Both relationship (clinical preceptor and coach) are in the primary interest of professional and personal development of the trainee. All faculty coaches will recuse themselves of voting in the Clinical Competency Committees (CCC, required by all programs by the ACGME) for trainees in this program. This has precedent specifically in the Internal Medicine program where mentors on the CCC do not vote on their personal mentees, which is the model we will use in our coaching program.

E. Potential Scientific Problems:

There is no ability to blind the intervention due to the nature of the program. The evaluation will rely on self-reported outcome measures, although we have made effort to identify and utilize evidence-based and validated evaluation tools. We cannot control for selection bias in how volunteers choose to participate in the program.

F. Data Analysis Plan:

Statistical analysis will be performed in an intent-to-treat basis. We will utilize univariate statistics for characterization of the sampled group. Comparisons between the group MBI and SCS over time will be used with paired t-test. Independent group t-test will be used to compare groups if enrollment exceeds capacity, and we are able to randomly generate a control group and intervention group. A PRA will independently analyze the interview transcripts using a mixed inductive/deductive approach to theme analysis supported by ATLAS.ti software.

G. Summarize Knowledge to be Gained:

Our pilot results demonstrate that our innovative coaching program is highly effective in mitigating and preventing burnout among female identifying GME trainees at CU. Because of our program design and already existing online platform for content, Better Together is an easily scalable, feasible program to address this problem among GME trainees nationwide. A

particular strength of the BT program format is that the web based, group coaching model with asynchronous elements like self-study modules and written coaching allows for a dramatically larger population to be served compared to models that rely on 1:1 coaching. Additionally, this structure allows for coaching by certified physician coaches with specific experience in coaching GME trainees as opposed to what exists in the prior coaching literature: using volunteer faculty “coaches" who are not certified, or certified but non-physician coaches without the same degree of expertise or experience.

H. References:

1. Rock D, Schwartz J. A brain-based approach to coaching. of Coaching. 2006. https://researchportal.coachfederation.org/Document/Pdf/2886.pdf. 2. Rock D, Page LJ. Coaching with the Brain in Mind: Foundations for Practice. John Wiley & Sons; 2009. 3. Palamara K, Kauffman C, Chang Y, et al. Professional Development Coaching for Residents: Results of a 3-Year Positive Psychology Coaching Intervention. J Gen Intern Med. 2018;33(11):1842-1844.

4. Castillo B. Self Coaching 101. Futures Unlimited Coaching; 2008. 5. Mura A. Executive Coaching Summit I: Documenting the Emerging Field of Coaching in Organizations. International Journal of Coaching in Organizations. 2003;1:19-26. 6. Neff KD. Self-Compassion Scale. PsycTESTS Dataset. 2012. doi:10.1037/t10178-000 7. Villwock JA, Sobin LB, Koester LA, Harris TM. Impostor syndrome and burnout among American medical students: a pilot study. J Int Assoc Med Sci Educ. 2016;7:364-369. 8. Mantri S, Lawson JM, Wang Z, Koenig HG. Identifying Moral Injury in Healthcare Professionals: The Moral Injury Symptom Scale-HP. J Relig Health. 2020;59(5):2323-2340.

FINAL STUDY PROTOCOL

Protocol #: 22-0028

Project Title: Better Together Physician Coaching: An Innovative Solution to Medical Trainee Burnout

Principal Investigator: Dr. Tyra Fainstad
Dr. Fainstad: tyra.fainstad@cuanschutz.edu, 720-480-4970

Version Date: June 27th, 2022

I. Hypotheses and Specific Aims: Our HYPOTHESIS is that Better Together, a 4-month, web-based positive psychology coaching program intervention for female residents when expanded to GME programs nationally, will result in decreased female trainee burnout.

- Aim 1: Prepare to expand the Better Together program by training and hiring a cohort of diverse physician coaches
- Aim 2: Implement Better Together in 10+ GME programs to serve 1000+ trainee participants with deliberate inclusion of institutions with diverse GME trainee populations serving geographically rural and/or medically underserved areas.
- Aim 3: Assess our outcomes: primary: reduce burnout as measured by the Maslach Burnout Index (goal: 10% relative improvement), and secondary: self-compassion, imposter syndrome, moral injury, and flourishing. We will also analyze outcome generalizability and program feasibility at a national level and look qualitatively at participant experience to gain a richer understanding of how Better Together may help trainees, in particular underrepresented minorities.
- Aim 4: Advance the field of coaching in GME through innovation and dissemination of evidence-based approaches to GME trainee wellbeing.

II. Background and Significance:

Burnout refers to feelings of exhaustion, negativism, and reduced personal efficacy resulting from chronic workplace stress. In healthcare, burnout leads to increased medical errors, poorer patient care and negatively affects professional development and retention. Burnout is a growing problem that begins early in medical training. Women and those underrepresented in medicine (URM) experience a disproportionate amount of burnout likely due to the cognitive load required to manage microaggressions, stereotypes, and harmful socially adopted narratives around efficacy. Professional coaching is a metacognition tool with a sustainable positive effect on physician well-being but typically relies on expensive consultants or time-consuming faculty development, often making it infeasible for medical training programs to offer. To overcome this barrier, we created Better Together Physician Coaching (BT) a 4-month coaching program for women residents at the University of Colorado (CU). BT includes regular online group-coaching, written coaching, and weekly self-study modules delivered by physician life coaches (Co-PIs). A pilot randomized controlled trial (RCT) of 101 BT participants demonstrated a statistically significant improvement in burnout, self-compassion, and imposter syndrome in the intervention group. This project will scale Better Together up to a national level and evaluated with an RCT mirroring our pilot in 10+ graduate medical education (GME) programs for 1000+ participants coordinated and evaluated by our CU team.

III. Preliminary Studies/Progress Report:

Within the past year, Drs. Adrienne Mann and Tyra Fainstad (AM, TF) created and implemented Better Together Physician Coaching (Better Together, or BT) at the University of Colorado School of Medicine. BT is a coaching program for female identifying GME trainees at CU and its affiliate hospitals (Denver Health, Rocky Mountain Regional VA Medical Center, and The Children's Hospital). Using coaching techniques, BT challenges long-held paradigms fostered by medical training. BT aims primarily to reduce burnout as measured by the MBI with secondary aims to increase self-compassion, reduce imposter syndrome and decrease moral injury among residents who identify as women.

AM and TF are both certified coaches through The Life Coach School™, a training institution for thought-based coaching. This type of coaching focuses on thoughts and beliefs. It combines a cognitive behavioral therapy (CBT) model with mindfulness-based awareness and integrates theories of acceptance and commitment therapy (ACT), nonattachment, and radical questioning from Socratic and Greek philosophies. BT delivers a robust coaching experience via a 6-month web-based, group-coaching model. This novel program allows residents to participate as actively as they are inclined and able, offering flexibility via multiple modalities of coaching: twice weekly group coaching calls, unlimited anonymous written coaching, and weekly self-study modules that are housed on a secure members-only website.

To study the BT program, the Co-PIs received institutional support from the CU Department of Medicine to conduct a pilot randomized controlled trial (RCT), which included support for professional research assistants to both implement and evaluate the program. A convenience sample of 101 female-identifying CU GME trainees from 12 specialties (IM, Family Medicine, Otolaryngology, Pediatrics, OBGYN, General Surgery, Emergency Medicine, Dermatology, Psychiatry, Medicine-Pediatrics, Pathology, and Neurology) was recruited and randomized to receive the 6-month Better Together Program or no-intervention from January-June 2021. The median participant age was 29 years, and all were female-identifying. Of the 101 participants, 33 (32.7%) were PGY-1, 43 (42.6%) were PGY-2, 18 (17.8%) were PGY-3, and 7 (7%) PGY-4 or greater. Nineteen (19%) of participants were in a surgical residency specialty (general surgery, OBGYN, otolaryngology). There were no significant differences in these characteristics between the intervention and control groups at baseline.

All participants completed a pre-survey assessing burnout with the MBI which defines burnout by three subscales (Emotional Exhaustion (EE), Depersonalization (DP), and Personal Accomplishment (PA) as described above). Each item is a 7-point Likert-type question with a frequency response scale ranging from 0 = never to 6 = every day. Higher scores on the EE and DP, and lower scores on the PA subscales indicate greater burnout. Secondary outcomes included Self-compassion with Self-Compassion Scale Short-form (SCSS) where higher scores indicate greater self-compassion,[30] the Young Imposter Syndrome Scale (YISS) where a score of more than 5 out of 8 points is diagnostic for Imposter Syndrome[31] and the Moral Injury Symptom Scale (MISS) (score 10-100 points) where higher scores equal more moral injury.[32]

Participants were randomized to the intervention group, (N=50) or control group (N= 51). The intervention group received the 6-month BT coaching program. The control group received the usual wellbeing curriculum provided by their training program, but no BT intervention. Within the BT coaching group, the most frequent topics of coaching included feedback reception, professional appearance, approval-addiction, deficit hiding, balancing motherhood with residency, and low self-confidence. Participants engaged in the live coaching sessions with curiosity, vulnerability, and authenticity, often bringing highly personal and emotional issues to the sessions and supporting each other through encouragement in the Zoom chat function.

From the pilot RCT, we found that at baseline over half of all participants were experiencing moderate or high burnout, consistent with national data. Participants were also experiencing low-moderate self-compassion (mean = 33.6 out of 60; SD=7.17); imposter syndrome (mean = 5.4 out of 8, where score of 5+ is diagnostic for imposter syndrome; SD=2.13); and moderate moral injury (mean =42.02 out of 100; SD=11.08). At the end of 6 months of coaching, a post-survey was offered to both the intervention and control groups. Of the 101 initial survey respondents, 79 responded to the post-survey (78%). A t-test was used to compare the change in subscale score means from baseline to 6 months in the BT coaching versus control groups for the primary and secondary outcomes.

The results on the MBI showed a statistically significant decrease in the emotional exhaustion (EE) dimension of burnout in the intervention group (p=0.03), and the DP and PA components of burnout both trended toward improvement. Self-compassion improved significantly in the intervention group compared to the control group, and imposter syndrome scores improved in the BT coaching group from 5.4 to 4.2 (p=0.01), effectively improving mean scores out of the range for imposter syndrome in this group. Moral injury also trended towards an improvement in the coaching vs control group from baseline to 6 months (40.7 to 35.6 versus 43.7 to 41.7 in intervention vs control, mean difference -3.84, p 0.10), but was not statistically significant.

Based on these promising findings in our pilot RCT, we propose scaling Better Together to a national level to address the unmet need in GME programs to reduce burnout. We have applied for HRSA grant funding (pending) which will allow us to plan, implement, and study a multi-institutional RCT to serve more female identifying trainees with a focus on those who are URM and practicing in medically underserved communities nationwide. In addition to adding an evidence-based innovative program to improve trainee burnout and wellness, this expanded trial will examine its generalizability and the feasibility of a model in which new coaches are trained to implement BT through a train-the-trainer model. We aim to enroll GME programs with geographic and ethnic diversity as well as representative URM trainees to understand the impact of coaching in this vulnerable population.

We are iteratively improving the intervention to optimize the content, duration, timing, and implementation. After three rounds of the 6-month program at CU, we have made the decision to condense the coaching material to 4 months instead. This is based on both participant and coach feedback around program flow and engagement. Our iterative experience with the coaching program has allowed for real-time improvements, including this one. To be clear, we are not changing or removing any part of the program’s content, only condensing certain months’ content into a shorter time. All studied content will still be available to participants to interact with asynchronously, but the timeline of presentation will shift to four months.

IV. Research Methods

A. Outcome Measure(s):

The primary outcome measure will include measures of burnout using the Maslach Burnout Inventory (MBI). Additional outcomes will include measures of self-compassion, imposter syndrome, moral injury, and flourishing as well as participation (administrative data). All data collection will be done in an aggregated format and in a confidential manner.

B. Description of Population to be Enrolled:

We will use convenience sampling to recruit an average of 100 trainee participants per GME program site, for a total of 1000+ trainee participants across up to 30 GME programs. Confirmed sites are included in the attached document with a site table. Inclusion criteria are: GME trainees (residents and fellows) who identify as women, trans-women, transfeminine, gender nonconforming, non-binary, and gender queer, who have at least 1 year left in their training program. The rationale for this last criterion is to ensure enrolled trainees will have time to complete the program prior to graduation. Enrollment in the program will be entirely voluntary and trainees can cease enrollment at any time.

C. Study Design and Research Methods

This randomized controlled trial will offer the intervention group the intervention of BT coaching during the first 4-months (September 1st 2022- December 23rd 2022) while the control group, or "waitlist group" will receive no intervention aside from the usual wellness activities of their institution. The control group will receive coaching from January 30th-May 30th 2023.

After recruitment and enrollment, all participants will be offered surveys containing the following validated indices to measure outcomes. Then they will be randomized, and after offered the same survey at the end of the first 4 months (Dec 23rd, 2022).

Primary Outcome Measures:

1. Burnout as defined by the Maslach Burnout Inventory (MBI) [Time Frame: pretest will occur prior to the intervention and post test will occur after the 4-month intervention. A post-post test will occur at 4 and 12 months after the intervention is done.]

   The Maslach burnout inventory (MBI) is a 22-item measurement of worker burnout which assesses emotional exhaustion (EE), depersonalization (DP), and personal fulfillment (PF) domains. Possible scores range from 0-6 on a Likert scale for each item. Scores of EE ≥ 27 points, DP ≥ 10, and PF <33 would indicate a high degree of burnout. Scores of EE≤18 points, DP≤5 points, and PF≥40 points would indicate a low degree of burnout.

Secondary Outcome Measures:

1. Self-Compassion as defined by Neff's Self Compassion Score Short Form (SCS-SF) [Time Frame: pretest will occur prior to the intervention and post test will occur after the 4 month intervention. A post-post test will occur 4 and 12 months after the intervention is done.]

   Neff's Self Compassion Score Short Form (SCS-SF) is a 12-item measurement of self-compassion. Possible scores range from 0-6 on a Likert scale for each item, where the higher scale scores indicate greater self-compassion. Scores of 1.0- 2.49 are considered to be low, between 2.5-3.5 to be moderate, and 3.51-5.0 to be high.

2. Moral Injury as defined by the Moral Injury Symptom Scale for Health Professions (MISS-HP) [Time Frame: pretest will occur prior to the intervention and post test will occur after the 4 month intervention. A post-post test will occur 4 and 12 months after the intervention is done.]
   Moral Injury Symptom Scale for Health Professions (MISS-HP) is a 10-item measurement of moral injury. Possible scores range from 0-5 on a Likert scale for each item, where the higher scale scores indicate greater moral injury. Scores >35(on a possible score range of 10 to 100) are considered high for moral injury symptoms

causing moderate to extreme problems with family, social, and occupational functioning.

3. Imposter Syndrome as defined by Young's Imposter Syndrome Symptoms Scale (YISS)
[Time Frame: pretest will occur prior to the intervention and post test will occur after the 4 month intervention. A post-post test will occur 4 and 12 months after the intervention is done.]
Young's Imposter Syndrome Symptoms Scale (YISS) is a 8-item measurement of imposter syndrome. Scoring is yes/no where a score of >5/8 is felt to be positive for imposter syndrome.

4. Flourishing as defined by the Secure Flourish Index (SFI)
[Time Frame: pretest will occur prior to the intervention and posttest will occur after the 4-month intervention. A post-post-test will occur 4 and 12 months after the intervention is done.]
The Secure Flourish Index (SFI) is a 12 item measurement of flourishing at work and includes the domains of (D1) happiness and life satisfaction; (D2) physical and mental health; (D3) meaning and purpose; (D4) character and virtue; and (D5) close social relationships plus 2 questions on having adequate stability as well as material and financial resources so that flourishing is likely to continue. Scores range from a low of 0 to a high of 120, though the secure flourishing scores are often reported as averages of the questions (rather than sums) so that all scores are on a scale of 0-10.

D. Description, Risks and Justification of Procedures and Data Collection Tools:

This is a low-risk intervention. Positive psychology coaching is not meant to replace or function as evaluation or medical care. We are using an internally developed survey in addition to multiple validated surveys that reflect the literature in similar programs. Any participant who demonstrates medically concerning issues will be immediately referred to appropriate evaluation. The content of the program is not intended for evaluation, so will not be shared with training programs. The participants will be instructed to maintain confidentiality of their peers' information, although given the group nature of this intervention, confidentiality cannot be assured. The faculty interests in their roles as clinical supervisors and coach are aligned in the promotion of trainee success. Both relationship (clinical preceptor and coach) are in the primary interest of professional and personal development of the trainee. All faculty coaches will recuse themselves of voting in the Clinical Competency Committees (CCC, required by all programs by the ACGME) for trainees in this program. This has precedent specifically in the Internal Medicine program where mentors on the CCC do not vote on their personal mentees, which is the model we will use in our coaching program.

E. Potential Scientific Problems:

There is no ability to blind the intervention due to the nature of the program. The evaluation will rely on self-reported outcome measures, although we have made effort to identify and utilize evidence-based and validated evaluation tools. We cannot control for selection bias in how volunteers choose to participate in the program.

F. Data Analysis Plan:

Statistical analysis will be performed in an intent-to-treat basis. We will utilize univariate statistics for characterization of the sampled group. Comparisons between the group MBI and SCS over time will be used with paired t-test. Independent group t-test will be used to compare groups if enrollment exceeds capacity, and we are able to randomly generate a control group and intervention

group. A PRA will independently analyze the interview transcripts using a mixed inductive/deductive approach to theme analysis supported by ATLAS.ti software.

G. Summarize Knowledge to be Gained:

Our pilot results demonstrate that our innovative coaching program is highly effective in mitigating and preventing burnout among female identifying GME trainees at CU. Because of our program design and already existing online platform for content, Better Together is an easily scalable, feasible program to address this problem among GME trainees nationwide. A particular strength of the BT program format is that the web based, group coaching model with asynchronous elements like self-study modules and written coaching allows for a dramatically larger population to be served compared to models that rely on 1:1 coaching. Additionally, this structure allows for coaching by certified physician coaches with specific experience in coaching GME trainees as opposed to what exists in the prior coaching literature: using volunteer faculty "coaches" who are not certified, or certified but non-physician coaches without the same degree of expertise or experience.

H. References:

1. Rock D, Schwartz J. A brain-based approach to coaching. *of Coaching.* 2006. https://researchportal.coachfederation.org/Document/Pdf/2886.pdf.
2. Rock D, Page LJ. *Coaching with the Brain in Mind: Foundations for Practice*. John Wiley & Sons; 2009.
3. Palamara K, Kauffman C, Chang Y, et al. Professional Development Coaching for Residents: Results of a 3-Year Positive Psychology Coaching Intervention. *J Gen Intern Med*. 2018;33(11):1842-1844.
4. Castillo B. *Self-Coaching 101*. Futures Unlimited Coaching; 2008.
5. Mura A. Executive Coaching Summit I: Documenting the Emerging Field of Coaching in Organizations. *International Journal of Coaching in Organizations*. 2003;1:19-26.
6. Neff KD. Self-Compassion Scale. *PsycTESTS Dataset*. 2012. doi:10.1037/t10178-000
7. Villwock JA, Sobin LB, Koester LA, Harris TM. Impostor syndrome and burnout among American medical students: a pilot study. *J Int Assoc Med Sci Educ*. 2016;7:364-369.
8. Mantri S, Lawson JM, Wang Z, Koenig HG. Identifying Moral Injury in Healthcare Professionals: The Moral Injury Symptom Scale-HP. *J Relig Health*. 2020;59(5):2323-2340.
9. Kelly-Hedrick,et al Measuring Flourishing Among Internal Medicine and Psychiatry Residents. J Grad Med Educ. 2020 Jun; 12(3): 312–319. doi: 10.4300/JGME-D-19-00793.1

Summary of Changes between Trial Protocol Versions:

1) Added the secondary outcome of Flourishing
    a. We became curious about the coaching program's ability to take participants from good to great, to impact those already doing well. The addition of this metric allows us to see if this tool is effective in the group that identifies as well also. The "Secure Flourishing Index" has been validated in health care professionals, and in medical trainees.

2) Switched from 2 "Rounds" to only 1 implementation phase of the RCT.
    a. We initially planned to phase in trainees to this RCT in 2 rounds. In 2021, we were not certain that we could offer the program to 1000 participants at once and did not want to risk under-delivering so modeled the trial in 2 rounds. However, as time progressed, and we successfully finished the pilot study and then had a few local cohorts go well with larger and larger numbers pf participants in BT, we decided that the program could easily support the larger numbers. We onboarded enough coaches to keep the coach:participant ratio the same as in our pilot even with over 1000 participants, so felt well-equipped to deliver the program in one RCT (1 round).

Statistical Analysis Plan (SAP)

Authors:

Tyra Fainstad, MD

Maria A. Woodward, MD MSc

Table of contents

1. Background
2. Study Design
    Fig 1. Study Schema
    2.1 Outcome measures
        2.1.1 Primary outcome measures
        2.1.2 Secondary outcome measures
    2.2 Determination of Sample Size
    2.3 Analysis timing
3. Study Conduct
    3.1 Randomization issues
4. Statistical Methods
    4.1 Analysis Populations
        4.1.1 Efficacy Analysis Populations
    4.2 Analysis of study conduct
    4.3 Analysis of treatment group comparability
    4.4 Efficacy analysis
    4.4.1 Primary Efficacy Endpoint
    4.4.2 Secondary Efficacy Endpoints

1. Background:

This Statistical Analysis Plan (SAP) provides details of the planned analyses and statistical methods for Study 22-0028: “Better Together Physician Coaching: An Innovative Solution to Medical Trainee Burnout.” The background for the study can be found in the study protocol.

2. Study Design:

This randomized, multicenter, study is designed to evaluate the efficacy of Better Together Physician Coaching in approximately 1000 female-identifying resident and fellow physicians in graduate medical education training programs across the country. Eligible participants were stratified by training site, specialty (surgical vs. non-surgical), and post graduate year (PGY) (PGY 1, 2, ≥3).

Eligible participants were randomized in a 1:1 ratio to either the intervention arm: receive the 4-month coaching program, Better Together Physician Coaching, or to a control arm: training as usual with no coaching program from September 1st 2022-December 31st 2022.

Participants randomized to the control arm were offered the coaching program after study completion and post-test data collection (Feb 1st 2023-May 31st 2023). Figure 1 illustrates the study design. The details for the study design can be found in the study protocol.

Figure 1: Study Schema

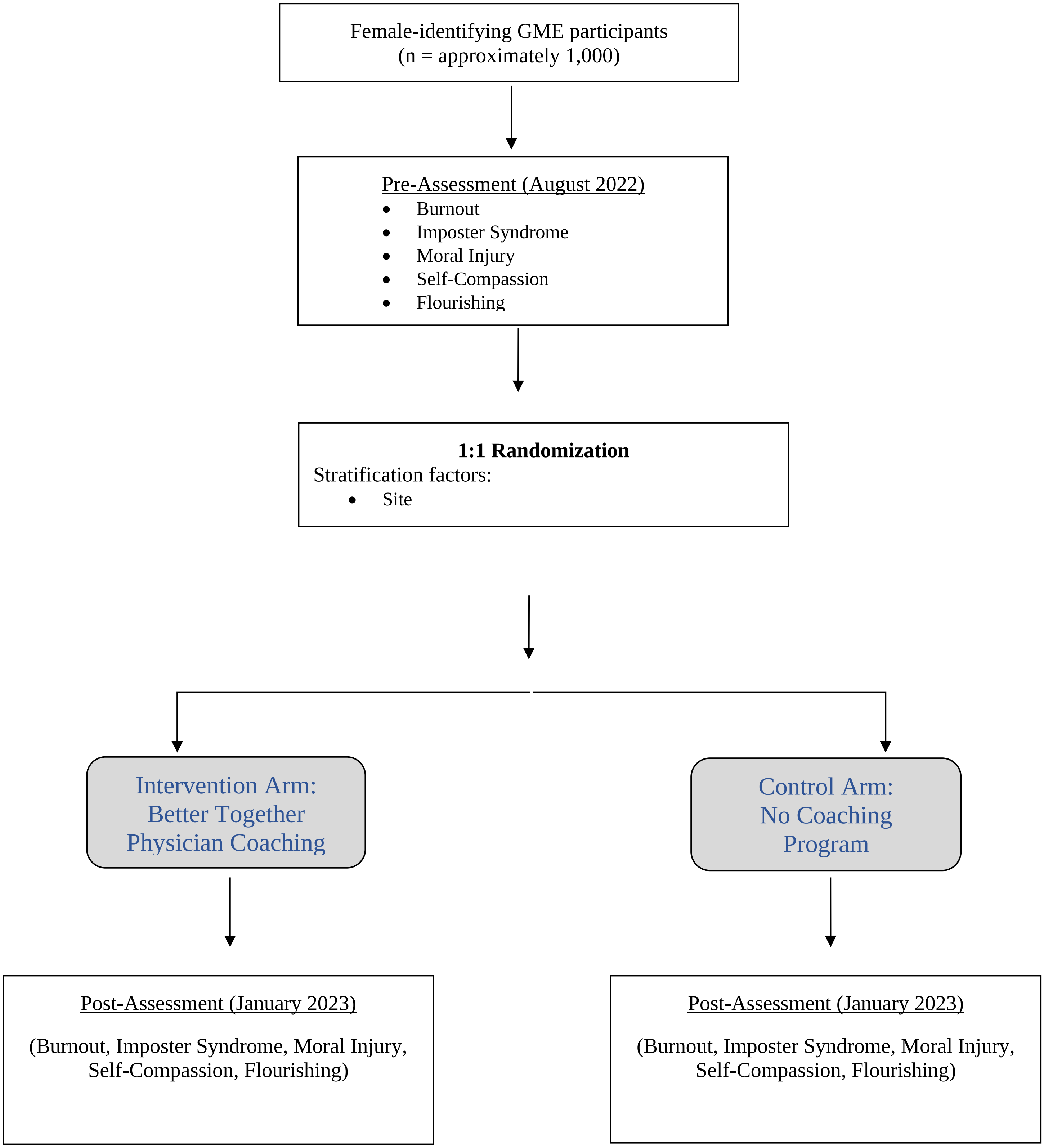

Participants are offered assessments at baseline and after the 4-month coaching program regardless of if they participated or level of engagement. Assessments are comprised of validated surveys measuring dimensions of physician distress (burnout, moral injury, imposter syndrome) and well-being (self-compassion, flourishing).

The primary outcome is burnout as assessed by the Maslach Burnout Inventory (MBI).

2.1 Outcome Measures

2.1.1 Primary Efficacy Outcome Measures

The primary outcome measure for this study is the Maslach Burnout Inventory (MBI). a 22-item measurement of worker burnout which assesses emotional exhaustion (EE), depersonalization (DP), and personal accomplishment (PA) domains. Possible scores range from 0-6 on a Likert scale for each item. Scores of EE ≥ 27 points, DP ≥ 10, and PA <33 would indicate a high degree of burnout. Scores of EE≤18 points, DP≤5 points, and PA≥40 points would indicate a low degree of burnout.

Burnout is defined using common thresholds to indicate the presence of burnout (high EE or high DP).

2.1.2 Secondary Efficacy Outcome Measures

The secondary outcomes measures for the study are the following:

- Impostor Syndrome: as defined by Young's Imposter Syndrome Symptoms Scale (YISS), an 8-item measurement of imposter syndrome. Scoring is yes/no and a score of >5/8 is considered positive for imposter syndrome.
- Moral Injury: as defined by the Moral Injury Symptom Scale for Health Professions (MISS-HP) is a 10-item measurement of moral injury. Possible scores range from 0-5 on a Likert scale for each item, where the higher scale scores indicate greater moral injury. Scores >35(on a possible score range of 10 to 100) are considered high for moral injury symptoms causing moderate to extreme problems with family, social, and occupational functioning.
- Self-Compassion: as defined by Neff's Self Compassion Score Short Form (SCS-SF) is a 12-item measurement of self-compassion. Possible scores range from 0-6 on a Likert scale for each item, where the higher scale scores indicate greater self-compassion. Scores of 1.0- 2.49 are considered to be low, between 2.5-3.5 to be moderate, and 3.51-5.0 to be high.
- Flourishing: as defined by The Secure Flourish Index (SFI) is a 12-item measurement of flourishing at work and includes the domains of (D1) happiness and life satisfaction; (D2) physical and mental health; (D3) meaning and purpose; (D4) character and virtue; and (D5) close social relationships plus 2 questions on having adequate stability as well as material and financial resources so that flourishing is likely to continue. Scores range from a low of 0 to a high of 120, though the secure flourishing scores are often reported as averages of the questions (rather than sums) so that all scores are on a scale of 0-10.

2.2 Determination of Sample Size

We assume a sample size of 1000 participants, based on an average expected enrollment of 250 participants from 4 sites. With an anticipated 20% drop out or loss-to-follow-up, we present a power calculation for a sample size of 800 participants (400 per arm). We conservatively assume that there is no change in the pre- and post-scores for the control arm and use estimates for the standard deviation (SD) of the change in scores from our pilot data. With 80% power (two-sided, alpha=0.05) we can detect a standardized effect size (SD=1) of 0.2, which is a small effect size by Cohen's classification.26 For the outcome of PA (SD of change in PA = 4.9), this corresponds to an increase in the change in PA between the intervention and control arm of 1.0 point (34.7 pre-coaching to 35.7 post-coaching in the intervention arm). For the outcome of DP (SD of change in DP = 8.6), this corresponds to a detectable decrease of 1.7 points (11.0 to 9.3). For the outcome of EE (SD of change in EE = 8.6), this corresponds to a detectable decrease of -1.7 points (27.1 to 25.4).

For all hypothesis testing performed, a p-value of less than 0.05 will be considered to indicate statistical significance.

2.3 Analysis Timing

No interim analyses are planned for the primary and secondary outcomes in this study. The final analysis for the primary comparison of the intervention arm and control arm will be conducted after the conclusion of the 4-month coaching program in January of 2023. The final analysis is expected to occur approximately 2 months after the final post-test is obtained.

3. Study Conduct

3.1 Randomization issues

Randomization to the intervention and control arms will occur using a computer-generated permutated-block randomization method. Randomization will be stratified by the following factors:

- Site

4. Statistical Methods

The analyses described in this SAP will supersede those specified in Protocol 22-0028 for the purposes of regulatory filing.

4.1 Analysis Populations

4.1.1 Efficacy Analysis Populations

The randomized population or ITT population is defined as all randomized participants, regardless of receipt of the assigned treatment.

4.2 Analysis of study conduct

Study enrollment, major protocol deviations including major deviations of inclusion or exclusion criteria, and reasons for study termination will be summarized overall and by intervention arm.

4.3 Analysis of treatment group comparability

Demographic characteristics, such as age, race, ethnicity, baseline disease characteristics (e.g., Maslach Burnout Inventory scale scores), and stratification factors will be summarized by intervention arms for the ITT populations. Descriptive statistics (mean, median, SD, and range) will be presented for continuous data, and frequencies and percentages will be presented for categorical data.

4.4 Efficacy analysis

Participants will be grouped for efficacy analyses in accordance with the intervention arm assigned at randomization, whether or not the coaching program was received. The primary analysis will be by intention-to-treat. Differences between groups will be expressed as relative risks and 95% confidence intervals.

The odds ratios are estimated from the intention to treat analysis using logistic mixed-effects regression models. The ORs represent the odds of burnout and impostor syndrome at follow-up in the intervention arm relative to the non-intervention arm (the reference group). Values below 1 indicate that participants in the intervention arm had lower odds of burnout and/or impostor syndrome at follow-up compared to the non-intervention arm. The models were of the form:

logit(probability of outcome) = beta0 + beta1*period + beta2*treatment + beta3*(period*treatment) + random-effect intercept for participant

Using the models above, the predicted probability of the outcome was computed for each observation in the data in two counterfactual cases: when treatment was “intervention” and when treatment was “non-intervention”. Then it calculated the odds ratio between these two sets of predictions, followed by the population-average of these odds ratios. Standard errors and confidence intervals were calculated using the delta method.”

4.4.1 Primary Efficacy Endpoint

The primary efficacy endpoint is burnout as defined by the MBI.

4.4.2 Secondary Efficacy Endpoints

The secondary efficacy endpoints are imposter syndrome, moral injury, self-compassion and flourishing as described above.

References:

1. Houkes I, Winants Y, Twellaar M, Verdonk P. Development of burnout over time and the causal order of the three dimensions of burnout among male and female GPs. A three-wave panel study. BMC Public Health. 2011;11:240.

2. Lautenberger DM, Dandar VM. The state of women in academic medicine 2018-2019: exploring pathways to equity. Association of American Medical Colleges. 2020.

3. Spataro BM, Tilstra SA, Rubio DM, McNeil MA. The Toxicity of Self-Blame: Sex Differences in Burnout and Coping in Internal Medicine Trainees. J Womens Health. 2016;25(11):1147-1152.

4. Pearl R. Uncaring: How the Culture of Medicine Kills Doctors and Patients. PublicAffairs; 2021.

5. Lundin RM, Bashir K, Bullock A, et al. "I'd been like freaking out the whole night": exploring emotion regulation based on junior doctors' narratives. Adv Health Sci Educ Theory Pract. 2018;23(1):7-28.

6. Resnick M. THE EFFECT OF AFFECT: DECISION MAKING IN THE EMOTIONAL CONTEXT OF HEALTH CARE. In: ; 2012. doi:10.1518/HCS-2012.945289401.007

7. Kafetsios K, Anagnostopoulos F, Lempesis E, Valindra A. Doctors' emotion regulation and patient satisfaction: a social-functional perspective. Health Commun. 2014;29(2):205-214.

8. Meeks LM, Ramsey J, Lyons M, Spencer AL, Lee WW. Wellness and Work: Mixed Messages in Residency Training. J Gen Intern Med. 2019;34(7):1352-1355.

9. Dyrbye L, Shanafelt T. A narrative review on burnout experienced by medical students and residents. Med Educ. 2016;50(1):132-149.

10. Guck AJ, Buck K. Reducing clinician inefficiency and restoring meaning in practice: A professional coaching approach for family medicine residents. Int J Psychiatry Med. July 2021:912174211034624.

11. Dyrbye LN, Shanafelt TD, Gill PR, Satele DV, West CP. Effect of a Professional Coaching Intervention on the Well-being and Distress of Physicians: A Pilot Randomized Clinical Trial. JAMA Intern Med. August 2019. doi:10.1001/jamainternmed.2019.2425

12. Palamara K, Chu JT, Chang Y, et al. Who Benefits Most? A Multisite Study of Coaching and Resident Well-being. J Gen Intern Med. June 2021. doi:10.1007/s11606-021-06903-5

13. Palamara K, Kauffman C, Chang Y, et al. Professional Development Coaching for Residents: Results of a 3-Year Positive Psychology Coaching Intervention. J Gen Intern Med. 2018;33(11):1842-1844.

14. Solms L, van Vianen A, Koen J, et al. Turning the tide: a quasi-experimental study on a coaching intervention to reduce burn-out symptoms and foster personal resources among medical residents and specialists in the Netherlands. BMJ Open. 2021;11(1):e041708.

15. Gazelle G, Liebschutz JM, Riess H. Physician burnout: coaching a way out. J Gen Intern Med. 2015;30(4):508-513.

16. Dyrbye LN, Shanafelt TD, Gill PR, Satele DV, West CP. Effect of a Professional Coaching Intervention on the Well-being, and Distress of Physicians: A Pilot Randomized Clinical Trial. JAMA Intern Med. August 2019. doi:10.1001/jamainternmed.2019.2425

17. Palamara K, Chu JT, Chang Y, et al. Who Benefits Most? A Multisite Study of Coaching and Resident Well-being. J Gen Intern Med. June 2021. doi:10.1007/s11606-021-06903-5

18. Palamara K, Kauffman C, Chang Y, et al. Professional Development Coaching for Residents: Results of a 3-Year Positive Psychology Coaching Intervention. J Gen Intern Med. 2018;33(11):1842-1844.

19. Solms L, van Vianen A, Koen J, et al. Turning the tide: a quasi-experimental study on a coaching intervention to reduce burn-out symptoms and foster personal resources among medical residents and specialists in the Netherlands. BMJ Open. 2021;11(1):e041708.

20. de Lasson L, Just E, Stegeager N, Malling B. Professional identity formation in the transition from medical school to working life: a qualitative study of group-coaching courses for junior doctors. BMC Med Educ. 2016;16:165.

21. Malling B, de Lasson L, Just E, Stegeager N. How group coaching contributes to organizational understanding among newly graduated doctors. BMC Med Educ. 2020;20(1):193.

22. Maslach C, Jackson SE, Leiter MP, Schaufeli W, Schwab R. Maslach Burnout Inventory Manual 4th Edition. Mind Garden. Inc: Menlo Park, CA. 2016.

23. Villwock JA, Sobin LB, Koester LA, Harris TM. Impostor syndrome and burnout among American medical students: a pilot study. J Int Assoc Med Sci Educ. 2016;7:364-369.

24. Neff KD. The Development and Validation of a Scale to Measure Self-Compassion. Self-Identity. 2003;2(3):223-250.

25. Mantri S, Lawson JM, Wang Z, Koenig HG. Identifying Moral Injury in Healthcare Professionals: The Moral Injury Symptom Scale-HP. J Relig Health. 2020;59(5):2323-2340.
