# Supplementary material for: Online Well-Being Group Coaching Program for Women Physician Trainees: A Randomized Clinical Trial
Source: JAMA Netw Open. 2023 Oct 4;6(10):e2335541. doi: 10.1001/jamanetworkopen.2023.35541 (PMC10551770; doi:10.1001/jamanetworkopen.2023.35541)
Supplement: Supplement 2. — eTable 1. Site Participation eMethods. BT Coach Onboarding and Facets of BT Program eTable 2. Participant Characteristics at Baseline by Survey Completion eTable 3. Effect Sizes for Burnout and Impostor Syndrome eTable 4. Intention to Treat Sensitivity Analysis: Mean Change in Response From Baseline Visit, Estimated From Linear Regression Adjusted for Baseline Value [file jamanetwopen-e2335541-s002.pdf]

Supplemental Online Content

Mann A, Shah AN, Thibodeau PS, et al. Online well-being group coaching program for women physician trainees: a randomized clinical trial. *JAMA Netw. Open.* 2023;6(9):e2335541.
doi:10.1001/jamanetworkopen.2023.35541

eTable 1. Site Participation
eMethods. BT Coach Onboarding and Facets of BT Program
eTable 2. Paricipant Characteristics at Baseline by Survey Completion
eTable 3. Effect sizes for Burnout and Impostor Syndrome
eTable 4. Intention to Treat Sensitivity Analysis: Mean Change in Response From Baseline Visit, Estimated From Linear Regression Adjusted for Baseline Value

This supplemental material has been provided by the authors to give readers additional information about their work.

eTable 1. Site Participation

Site (State)	# of Participating trainees (% of total)
Site A (TX) Brooke Army Medical Center; San Antonio Uniformed Services Health Education Consortium	6 (0.59%)
Site B (NJ) Cooper Medical School of Rowan University; Cooper University Health Care and	23 (2.26%)
Site C (NC) East Carolina University Health Medical Center	16 (1.57%)
Site D (GA) Emory University School of Medicine	44 (4.33%)
Site E (MD) Johns Hopkins School of Medicine; Johns Hopkins University	92 (9.05%)
Site F (WA) Kadlec Regional Medical Center; Providence GME	1 (0.10%)
Site G (PA) Main Line Health System; Lankenau Medical Center	10 (0.98%)
Site H (AK) Providence Alaska Medical Center; Providence GME	13 (1.28%)
Site I (OR) Providence Portland Medical Center; Providence GME	22 (2.16%)
Site J (WA) Providence Sacred Heart Medical Center; Providence GME	16 (1.57%)
Site K (WA) Providence Saint Peter Hospital; Providence GME	3 (0.20%)
Site L (IL) Rush Medical College; Rush University	35 (3.44%)
Site M (CA) Providence St. Johns' Health Center; Providence GME	2 (0.20%)
Site N (CO) Saint Joseph Hospital; Intermountain Health	19 (1.87%)
Site O (WA) Swedish Medical Center; Providence GME	2 (0.20%)
Site P (MA) Tufts University School of Medicine; Tufts Medical Center	57 (5.60%)
Site Q (CA) University of California at San Diego School of Medicine	83 (8.16%)
Site R (CO) University of Colorado School of Medicine	126 (12.39%)
Site S (KY) University of Kentucky College of Medicine	32 (3.15%)
Site T (NM) University of New Mexico School of Medicine, University of New Mexico Health Science Center	46 (4.52%)
Site U (TX) University of Texas Health Science Center at San Antonio	69 (6.78%)
Site V (VT) University of Vermont Larner College of Medicine; University of Vermont Medical Center	12 (1.18%)
Site W (WA) University of Washington School of Medicine	106 (10.42%)

© 2023 Mann A et al. *JAMA Network Open.*

Site X (WA) Virginia Mason Franciscan Health	12 (1.18%)
Site Y (MO) Washington University School of Medicine in St. Louis	78 (7.67%)
Site Z (CT) Yale School of Medicine; Yale New Haven Health	92 (9.05%)

***** We requested the total number of GME trainees who self-reported as women from all of our sites, and 18 of 26 provided this information. Among these 18 programs, the total number of female-identifying GME trainees was 6,108. For the remainder of the programs (n = 8, 7 small community and 1 mid-sized academic center) we estimated an additional 318 trainees were eligible based on their total program size available on public websites, and national percentage of female trainees. This gave a total of 6,426.

eMethods. BT Coach Onboarding and Facets of BT Program

Better Together Coach Onboarding Process

To deliver the program at scale we onboarded >20 physician coaches from across the country, all physicians and all certified through the Life Coach School (see #2 below). Though BT coaches have all been certified through LCS, none are affiliated with the LCS in an ongoing way, specifically, none are currently employees or teachers for LCS. The LCS was chosen for it's thought based coaching methodology (rather than behavior based), technique is theory-informed, evidence-based, and algorithmic. Because of this, the coach who uses the LCS model delivers a reproducible and valid technique, which is not the case in many other programs. Additionally, most other coaching programs center on hour-long 1:1 coaching sessions, while LCS specifically trains to provide coaching in both written, and a virtual (zoom) group format as well as how to facilitate shorter sessions (10-15minuts), which are imperative to our group calls. Coach certification cost between $15,000-$20,000 for our coaches (prices have increased over time). Many of the BT coaches paid for this out of their own pocket (including AM and TF), however, some have been supported in full or part by their institutions. Each coach volunteered to host at least two coaching calls during the 4-month program on the Better Together platform with regular oversight and feedback from Drs. Mann and Fainstad to ensure internal reliability and quality of coaching. Though some of these coaches were faculty at participating sites, that was not a requirement. In the instance that a BT coach coincidentally supervised a participant in a clinical or educational setting, they recused themselves from any formal assessment to avoid potential conflict of interest.

To ensure quality and internal consistency of our coaches and coaching, we had the following criteria for each coach:

1) Must be a physician (MD or DO).

2) Must have graduated from the Life Coach School (LCS) certification program.

3) Must submit a sample coaching call which is reviewed by Drs. Mann and Fainstad, with provision of feedback with a standardized peer- observation feedback tool (available upon request). If their coaching sample was acceptable, the coach was invited to be added to our roster.

© 2023 Mann A et al. *JAMA Network Open.*

4) Must agree to receive self and peer-feedback on coaching. Once on our roster, coaches sign up to host coaching calls. These calls are observed intermittently with provision of feedback via a standardized peer-observation tool.

For ongoing fidelity/quality assurance of coaching, Drs. Fainstad and Mann observed and provided feedback for each coach at least once during the intervention and did this on a quarterly basis for each coach that works in BT in previous (local) iterations of this program. If a coach was not providing coaching consistent with the model and feedback framework, then underwent remediation with Drs. Fainstad and Mann until these metrics are met. All coaching calls were recorded and housed on a secure, members-only website.

BT Foundational Framework:

Certified physician coaches	Many coaching interventions use non-physician coach consultants or rely on volunteer physician faculty with variable degrees of faculty development training rather than formal certification.[11,19] Utilization of physician coaches allowed for contextual understanding of challenges of medical training and offered relatability and credibility to participants.
Group Coaching	Delivering coaching on a group call offers the same impact for the individual being coached with the additional benefit for all observing who can apply lessons learned and gain a sense of normalization of vulnerability and community.
Flexibility [24,25]	The asynchronous, multimodal content delivered by BT allows trainees to access material on-demand. The repository of recorded calls allows participants who miss a call to still reap the benefits. Having volunteer coaches across the country allowed us to offer a variety of call times to accommodate trainee schedule needs across 5 time zones.
Personalized Anonymity [24,25]	Participants could choose how, when and whether to show up on any facet of BT. Group calls were held in a webinar format, so the audience was completely anonymous. If they chose to join as a panelist and be coached live, they decided on their visibility (camera on or off, use of their name, etc.). They also had the option to be coached live in written format using the chat function. Offering an asynchronous anonymous ask-for-coaching written forum offered those who felt too exposed on live calls a space to write in and receive coaching in that form as well.
Data-driven	The BT program is evidence-based, and we employ a "Plan-Do-Study-Act" (PDSA) cycle (an iterative, four-stage problem-solving method used for improving a process)[1] after each coaching cohort in which we review the various coaching requests that arose in our participants and focus our curriculum and coach training around these areas specifically. The program is iteratively improved between each cycle based on participant feedback (eg: inclusion of a week on microaggressions and bias, and shortened length)

1) Taylor MJ, McNicholas C, Nicolay C, Darzi A, Bell D, Reed JE. Systematic review of the application of the plan-do-study-act method to improve quality in healthcare. *BMJ Qual Saf.* 2014;23(4):290-298.

Modalities of the Better Together Coaching Program:

Live Coaching Calls. The live coaching calls were facilitated by one of the certified physician coaches. Calls were hosted on the Zoom video-conferencing platform and were 60 minutes each. Getting coached on a live call was optional and voluntary: participants could raise their hand to

© 2023 Mann A et al. *JAMA Network Open.*

request to be coached while the other participants on the call observed. Coaching on a call could be received anonymously through the chat, Q&A function or verbally. Call audio recordings were published on a private podcast accessible only to participants so they could listen to any calls they were not able to attend live. Coaching calls were scheduled at a variety of days and times to serve participants in different time zones and with variable schedules.

"Ask-for-Coaching" Written Coaching. The secure website housed an online forum where participants could anonymously request coaching via written format. Responses to written coaching requests were posted to the forum within 1-business day by TF or AM. This asynchronous format allowed access to coaching around-the-clock and provided a kinesthetic learning format for participants to engage with the coaching material. Ask-for-Coaching could be accessed at any point throughout the 4-month intervention, and the coachee was not identifiable to other participants.

Worksheets and Webinars. At the start of each month of content, worksheets were released which accompanied each weekly theme (i.e., receiving feedback, setting goals, adopting a growth mindset, or defining work-life balance). The worksheets facilitate metacognitive techniques of awareness and processing feelings. The worksheets were voluntary and meant for independent self-study. Short 5–10-minute webinar videos corresponding to weekly content were released at the start of each month. The webinars were chalk-talk style didactics given by AM or TF to introduce weekly themes. As they may be watched repeatedly, and at any time, the webinars served as an asynchronous component of the program that participants engaged with at their own pace.

BT worksheet/webinar curriculum:

<u>Month 1: Coaching 101</u>
Week 1: Metacognition: Introduction to the coaching model
Week 2: Emotions: How to name and process an emotion
Week 3: Defining your Purpose: using values to create meaning
Week 4: Living your Purpose at Work: finding your power to mitigate moral injury

<u>Month 2: Feedback</u>
Week 1: Growth Mindset
Week 2: Welcoming Feedback: especially when it's hard to hear
Week 3: Bias and Microaggressions: accessing empowerment instead of burnout
Week 4: Being your Best at Work: showing up for yourself even when it's challenging

<u>Month 3: Imposter Syndrome</u>
Week 1: Imposter Syndrome: getting to know your inner critic
Week 2: Perfectionism: the stealthy self-saboteur
Week 3: Approval Addiction: why people pleasing doesn't work for you
Week 4: How to change: reframe your inner critic

<u>Month 4: Launching into the New You</u>
Week 1: Self-confidence: how it's different from arrogance and task-confidence
Week 2: Transitions: navigating through constant change
Week 3: Self-Compassion: tapping into your inner wisdom
Week 4: The New You- become your own best friend, for life

© 2023 Mann A et al. *JAMA Network Open.*

eTable 2. Participant Characteristics at Baseline by Survey Completion

			Completed		
**Variable**	**N**	**Overall**, N = 1,017[1] No./No. obs. (% not missing)	**Both surveys**, N = 412[1] No./No. obs. (%)	**Pre-survey only**, N = 606[1] No./No. obs. (%)	**p-value**[2]
**Age**	851	851 / 1,017 (83.6)	363 / 412 (88.1)	488 / 606 (80.5)	0.046
Mean (SD)		30.9 (4.0)	31.2 (3.9)	30.8 (4.0)	
Median (IQR)		30.0 (28.0, 33.0)	31.0 (29.0, 33.0)	30.0 (28.0, 33.0)	
Range		24.0 - 56.0	24.0 - 52.0	24.0 - 56.0	
**Post Graduate Year (PGY)**	1,000				<0.001
PGY1		207 / 1,000 (20.7)	61 / 410 (14.9)	146 / 590 (24.7)	
PGY2		198 / 1,000 (19.8)	80 / 410 (19.5)	118 / 590 (20.0)	
PGY3+		595 / 1,000 (59.5)	269 / 410 (65.6)	326 / 590 (55.3)	
**Specialty**	999				>0.9
Non-Surgical		813 / 999 (81.4)	333 / 410 (81.2)	480 / 589 (81.5)	
Surgical		186 / 999 (18.6)	77 / 410 (18.8)	109 / 589 (18.5)	
**Gender Identity**	1,017				0.019
Woman		959 / 1,017 (94.2)	397 / 412 (96.4)	562 / 606 (92.7)	

© 2023 Mann A et al. *JAMA Network Open.*

Man, Non-binary, not listed, other, prefer not to say		59 / 1,017 (0.2)	13 / 412 (0.5)	37 / 606 (0.06)	
**Gender different from sex assigned at birth?**	963				0.8
No		938 / 963 (97.4)	388 / 399 (97.2)	550 / 564 (97.5)	
Yes/prefer not to say		25 / 963 (2.5)	11 / 399 (2.8)	14 / 564 (2.3)	
**Race**	1,018				0.003
Asian		229 / 1,018 (22.5)	89 / 412 (21.6)	140 / 606 (23.1)	
American Indian and Alaskan Native		2 / 1,018 (0.2)	0 / 412 (0.0)	2 / 606 (0.3)	
Black or African American		52 / 1,018 (5.1)	17 / 412 (4.1)	35 / 606 (5.8)	
LatinX/Hispanic		83 / 1,018 (8.2)	24 / 412 (5.8)	59 / 606 (9.7)	
Multiracial		35 / 1,018 (3.4)	14 / 412 (3.4)	21 / 606 (3.5)	
Native Hawaiian and Other Pacific Islander		2 / 1,018 (0.2)	1 / 412 (0.2)	1 / 606 (0.2)	
Other		158 / 1,018 (15.5)	44 / 412 (10.7)	114 / 606 (18.8)	
White		540 / 1,018 (53.0)	247 / 412 (60.0)	293 / 606 (48.3)	
**Sexual orientation**	958				0.5
Heterosexual		843 / 958 (88.0)	356 / 399 (89.2)	487 / 559 (87.1)	
Homosexual		21 / 958 (2.2)	7 / 399 (1.8)	14 / 559 (2.5)	

© 2023 Mann A et al. *JAMA Network Open.*

Bisexual		68 / 958 (7.1)	24 / 399 (6.0)	44 / 559 (7.9)	
Not Listed		6 / 958 (0.6)	4 / 399 (1.0)	2 / 559 (0.4)	
Prefer not to say		20 / 958 (2.1)	8 / 399 (2.0)	12 / 559 (2.1)	

[1]n / N (%)
[2]Pearson's Chi-squared test; Wilcoxon rank sum test; Fisher's Exact Test for Count Data with simulated p-value (based on 2000 replicates); Fisher's exact test

© 2023 Mann A et al. *JAMA Network Open.*

eTable 3. Effect sizes for Burnout and Impostor Syndrome
Population-averaged (marginal) odds ratios, estimated from logistic mixed-effects models.

Outcome	OR (95% CI)	p
Probability of Burnout	0.47 (0.28 to 0.78)	0.004
Probability of Young Impostor	0.36 (0.21 to 0.62)	<0.001

eTable 4. Intention to Treat Sensitivity Analysis: Mean Change in Response From Baseline Visit, Estimated From Linear Regression Adjusted for Baseline Value

Outcome	Mean change from baseline (95% CI)	p
Burnout Emotional Exhaustion	-2.44 (-3.66 to -1.22)	<0.001
Burnout Personal Accomplishment	1.30 (0.47 to 2.12)	0.002
Burnout Depersonalization	-0.80 (-1.51 to -0.08)	0.029
Young Impostor Scale Total	-0.68 (-0.94 to -0.42)	<0.001
Moral Injury Scale	-3.27 (-4.92 to -1.61)	<0.001
Self-compassion Scale	2.61 (1.69 to 3.53)	<0.001
Flourishing Scale	0.32 (0.16 to 0.49)	<0.001

© 2023 Mann A et al. *JAMA Network Open.*
