# Supplementary material for: Online Well-Being Group Coaching Program for Women Physician Trainees: A Randomized Clinical Trial
Source: JAMA Netw Open. 2023 Oct 4;6(10):e2335541. doi: 10.1001/jamanetworkopen.2023.35541 (PMC10551770; doi:10.1001/jamanetworkopen.2023.35541)
Supplement: Supplement 3. — Data Sharing Statement [file jamanetwopen-e2335541-s003.pdf]

Data Sharing Statement

Data

Data available: Yes
Data types: Deidentified participant data
How to access data: tyra.fainstad@cuanschutz.edu
When available: With publication

Supporting Documents

Document types: Statistical/analytic code, Informed consent form
How to access documents: tyra.fainstad@cuanschutz.edu
When available: With publication

Additional Information

Who can access the data: researchers whose proposed use of the data has been approved
Types of analyses: for a specified research purpose
Mechanisms of data availability: with a signed data access agreement
